# Human dental pulp stem cells derived extracellular matrix promotes mineralization via Hippo and Wnt pathways

**DOI:** 10.1038/s41598-024-56845-1

**Published:** 2024-03-21

**Authors:** Chatvadee Kornsuthisopon, Nunthawan Nowwarote, Ajjima Chansaenroj, Suphalak Photichailert, Sunisa Rochanavibhata, Nuttha Klincumhom, Stephane Petit, Florent Dingli, Damarys Loew, Benjamin P. J. Fournier, Thanaphum Osathanon

**Affiliations:** 1 Center of Excellence for Dental Stem Cell Biology and Department of Anatomy, Faculty of Dentistry, Chulalongkorn University, 34 Henri-Dunant Rd. Pathumwan, Bangkok, 10330 Thailand; 2 Centre de Recherche des Cordeliers, Université Paris Cité, Sorbonne Université, INSERM UMR1138, Molecular Oral Pathophysiology, 75006 Paris, France; 3 Department of Oral Biology, Faculty of Dentistry, Université Paris Cité, 75006 Paris, France; 4 Department of Oral and Maxillofacial Surgery, Faculty of Dentistry, Chulalongkorn University, Bangkok, 10330 Thailand; 5 Institut Curie, Centre de Recherche, Laboratoire de Spectrométrie de Masse Protéomique, PSL Research University, 26 Rue d’Ulm, 75248 Cedex 05 Paris, France

**Keywords:** Stem-cell research, Cell signalling

## Abstract

Extracellular matrix (ECM) is an intricate structure providing the microenvironment niche that influences stem cell differentiation. This study aimed to investigate the efficacy of decellularized ECM derived from human dental pulp stem cells (dECM_DPSCs) and gingival-derived mesenchymal stem cells (dECM_GSCs) as an inductive scaffold for osteogenic differentiation of GSCs. The proteomic analysis demonstrated that common and signature matrisome proteins from dECM_DPSCs and dECM_GSCs were related to osteogenesis/osteogenic differentiation. RNA sequencing data from GSCs reseeded on dECM_DPSCs revealed that dECM_DPSCs upregulated genes related to the Hippo and Wnt signaling pathways in GSCs. In the inhibitor experiments, results revealed that dECM_DPSCs superiorly promoted GSCs osteogenic differentiation, mainly mediated through Hippo and Wnt signaling. The present study emphasizes the promising translational application of dECM_DPSCs as a bio-scaffold rich in favorable regenerative microenvironment for tissue engineering.

## Introduction

Extracellular matrix (ECM) functions as a physical supporting structure for cells^1^. ECM directly communicates with cells through cell surface receptors, mainly integrins, which initiate downstream intracellular signaling and control various cellular functions^2^. Additionally, ECM regulates stem cell activity by acting as a reservoir and mediating the release of growth factors^3,4^. Further, ECM stiffness is crucial in mechanotransduction, facilitating cell migration, cell cycle progression, and cell fate determination^5^. Overall, ECM's intricate regulation of cellular behavior underscores its importance in stem cell biology and tissue regeneration.

Since biomimetic exogenous scaffolds are often lacking, the ECM becomes even more critical as it mimics the natural biological environment and supports cellular processes that promote tissue regeneration^6–9^. ECM can be obtained through cultured cell ECM production and decellularization^10^. The decellularization process preserves ECM proteins while cellular components are removed to avoid immune reactions^11^. Decellularized ECM (dECM) of oral tissues enhances constructive remodeling for tissue engineering applications when dECM serves as a scaffold. dECM derived from dental pulp stem cells that were cultured in an osteogenic induction medium (OM-dECM_DPSCs) contains mineralization-associated factors that could promote osteogenic differentiation of gingival-derived mesenchymal stem cells (GSCs) without chemical cues^10^. However, specific pathways of osteogenic regulation of ECM derived from different cell types remain elusive. The present study aims to investigate the composition and function of dECM from DPSCs and GSCs as well as to examine regulatory signaling pathways. Overall, this research aims to provide insights into the complex cell-ECM interactions with potential implications for tissue engineering and regenerative medicine.

## Results

### Cell characterization

**Figure 1:**
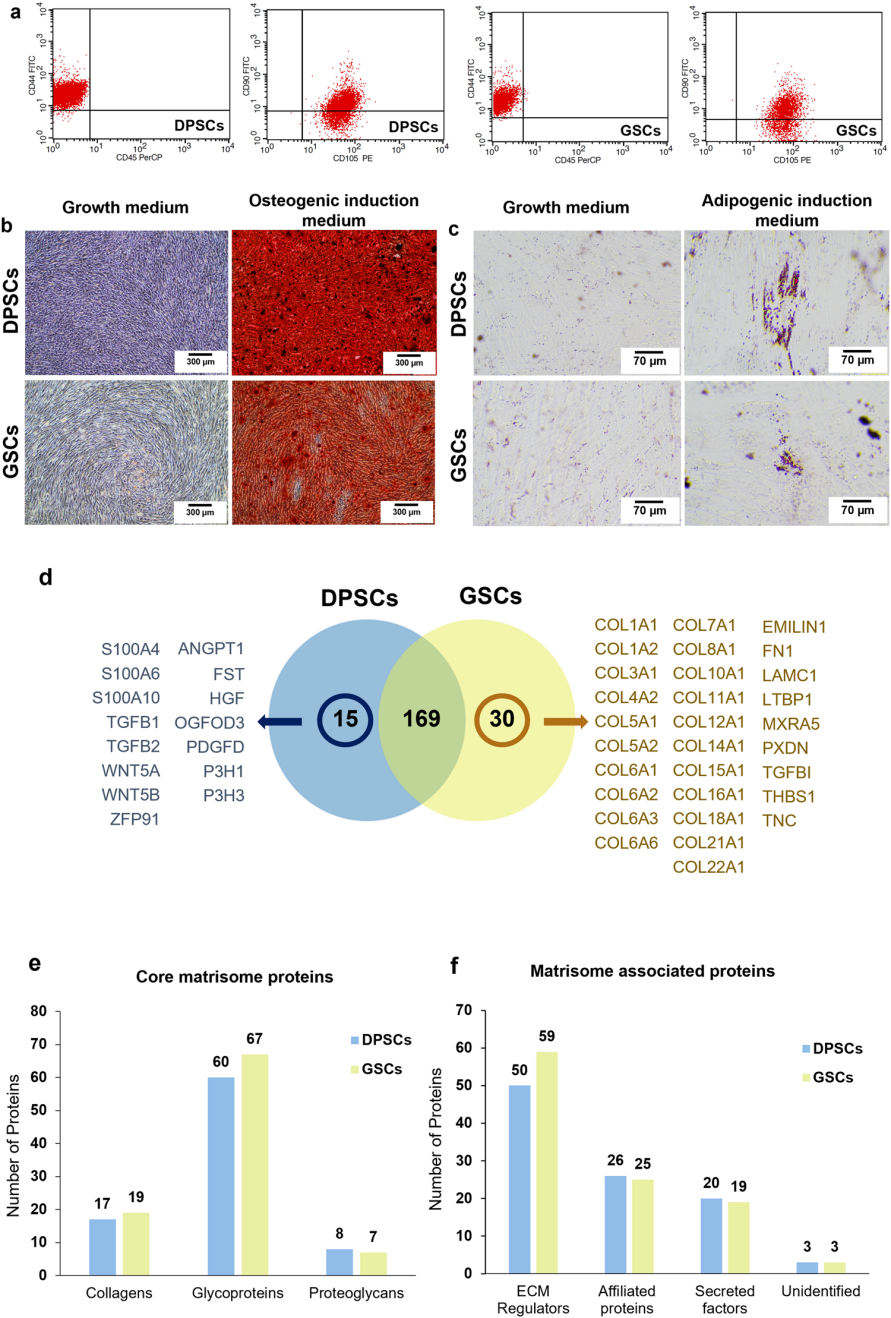
Characterization of the cells isolated from dental pulp tissues and gingiva. (**a**) Evaluation of stem cell surface markers using flow cytometry. Multi-lineage differentiation potential toward (**b**) osteogenic and (**c**) adipogenic lineage. Proteomic profile of decellularized extracellular matrix derived from human dental pulp stem cells (dECM_DPSCs) and gingival-derived mesenchymal stem cells (dECM_GSCs) demonstrated that the signature proteins of dECM_DPSCs and dECM_GSCs were members of the osteogenic-associated pathways. (**d**) Venn diagram of quantified common proteins and unique proteins from dECM_DPSCs and dECM_GSCs. The analyzes were performed using an interactive Venn diagram viewer^47^. All proteins of each cell were analyzed and classified as (**e**) core matrisome and (**f**) matrisome associated proteins, respectively, using the human matrisome database downloaded from http://matrisomeproject.mit.edu/other-resources/human-matrisome/^48^.

Surface protein analyzed by flow cytometry showed that both DPSCs and GSCs were positively stained for mesenchymal stem cell-related surface markers (CD44, CD90, and CD105) and negatively stained for hematopoietic cell marker CD45 (Fig. 1a). Mineral deposits were observed by Alizarin Red S (ARS) staining in both cell types after being cultured in an osteogenic induction medium for 14 days (Fig. 1b). The cells cultured in an adipogenic induction medium for 16 days demonstrated an increased intracellular lipid accumulation as stained by oil red o staining (Fig. 1c). These results confirmed that the isolated DPSCs and GSCs used in the subsequent experiments were mesenchymal stem cells.

### Matrisome profile of dECM_DPSCs and dECM_GSCs

The normal ECM (N-ECM) from both cell types was decellularized. Quantitative mass spectrometry analysis was performed by comparing the triplicate of each condition. Results displayed 214 quantified proteins belonging to the matrisome proteins with at least 3 total peptides (Supplementary Table 1). The Venn diagram presented all the quantified common matrisome proteins (169 proteins) and the unique proteins from dECM_DPSCs (15 proteins) and dECM_GSCs (30 proteins) (Fig. 1d).

The number of classified matrisome differed slightly between dECM from each cell type. A total of 85 and 93 proteins from core matrisome proteins were detected in dECM_DPSCs and dECM_GSCs, respectively (Fig. 1e). As for the matrisome-associated proteins (i.e., ECM regulators, ECM-affiliated proteins, secreted factors, and unidentified proteins), dECM_DPSCs had 99 proteins, while dECM_GSCs had 106 proteins (Fig. 1f). The major core matrisome and matrisome-associated proteins in both cell types were glycoproteins and ECM regulators, respectively.

### dECM_from DPSCs and dECM_from GSCs exhibited matrisome proteins related to calcium ion binding

**Figure 2:**
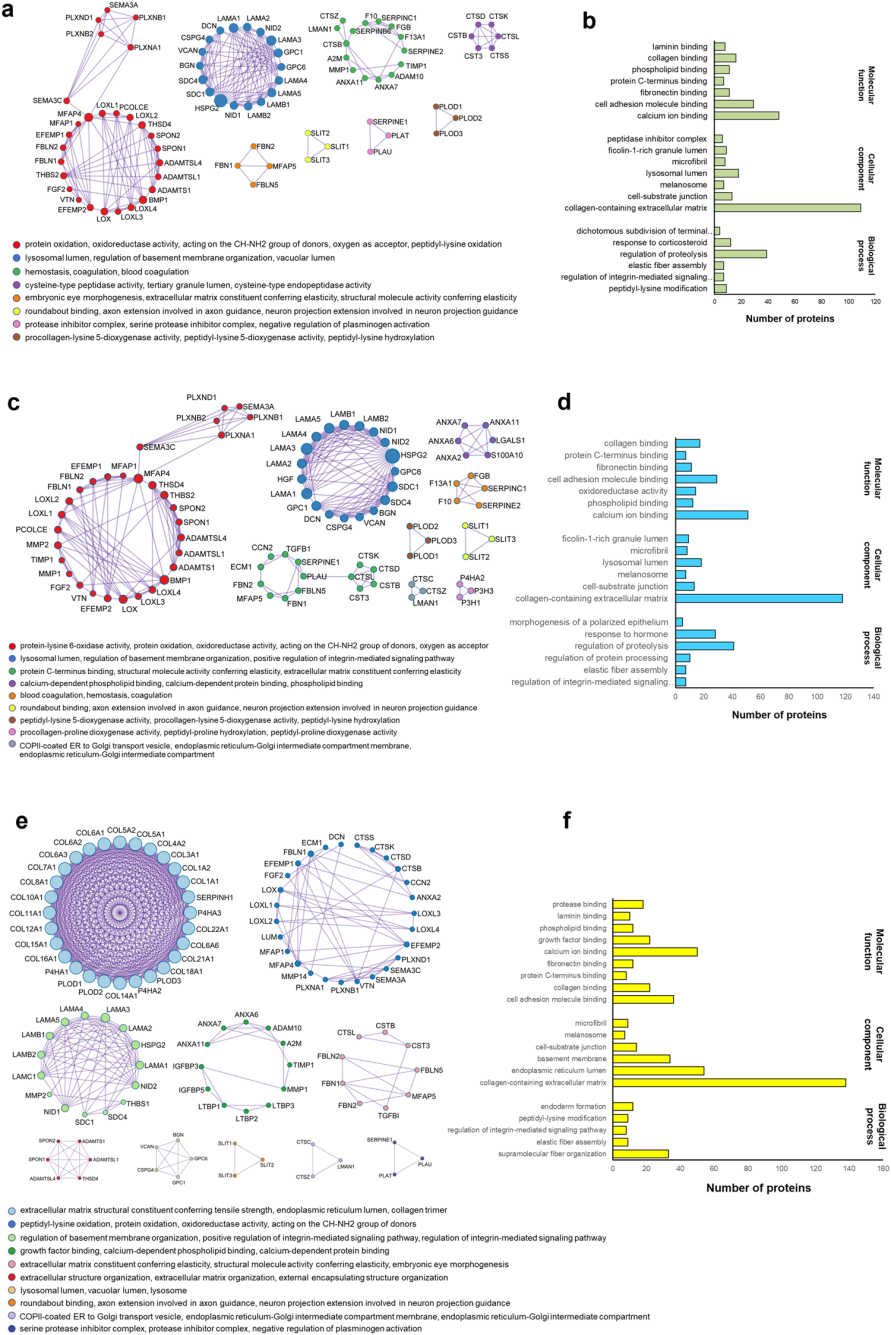
dECM_DPSCs and dECM_GSCs exhibited matrisome protein related to calcium ion binding. (**a**) Network analysis of protein–protein interaction (PPI) following the Molecular Complex Detection (MCODE) components and (**b**) gene ontology analysis of the overlapped matrisome proteins of dECM_DPSCs and dECM_GSCs. (**c**) Network analysis of PPI following the MCODE components and (**d**) gene ontology analysis of matrisome proteins of dECM_DPSCs. (**e**) Network analysis of PPI following the MCODE components and (**f**) gene ontology analysis of matrisome proteins of dECM_GSCs. The analyzes were performed using Metascape^49^.

Metascape analysis was utilized to present the protein–protein interaction of common matrisome proteins. The overlapped matrisome proteins of dECM_DPSCs and dECM_GSCs were mainly associated with protein oxidation, regulation of basement membrane organization, and homeostasis (Fig. 2a). The gene ontology enrichment analysis was carried out with the following gene ontology (GO) sources: GO Biological Process, GO Cellular Component, and GO Molecular Functions. The main functions of the common matrisome classified by GO sources were the regulation of proteolysis, collagen-containing ECM, and calcium ion binding, respectively (Fig. 2b).

We next analyzed the DPSCs matrisome. The Protein–protein interaction (PPI) of dECM_DPSCs matrisome proteins was primarily associated with proteins oxidation, regulation of basement membrane organization, and ECM constituent conferring elasticity (Fig. 2c). The molecular function of these proteins was mainly calcium-ion binding, while collagen containing ECM was their main cellular component (Fig. 2d).

GSCs matrisome showed that the PPI was principally involved with ECM structural constituent conferring tensile strength, peptidyl-lysine hydroxylation, and regulation of basement membrane organization (integrin) (Fig. 2e). The highest number of proteins in each GO source (biological process, cellular component, and molecular function) was involved with supramolecular fiber organization, collagen-containing ECM, and calcium ion binding, respectively (Fig. 2f).

### dECM_DPSCs’ and dECM_GSCs’ signature proteins were members of the mineralization-associated pathway and structural proteins

dECM_DPSCs had 15 signature (unique) proteins consisting of TGFB2, S100A6, WNT5A, TGFB1, S100A4, HGF, FST, S100A10, WNT5B, ZFP91, PDGFD, ANGPT1, P3H1, P3H3, and OGFOD3 (Fig. 1d). Meanwhile, dECM_GSCs demonstrated 30 signature proteins classified as collagen and non-collagen cluster. Twenty-one proteins were mainly presented in the collagen cluster. Non-collagen cluster contained FN1, TNC, MXRA5, EMILIN1, THBS1, TGFB1, PXDN, and LAMC1 (Fig. 1d). Therefore, the signature proteins of dECM_DPSCs pointed out in two key osteogenic-related pathways: TGF-β and Wnt signaling pathways.

### GSCs on dECM_DPSCs expressed genes involved TGF-β, Hippo, and Wnt signaling pathways

**Figure 3:**
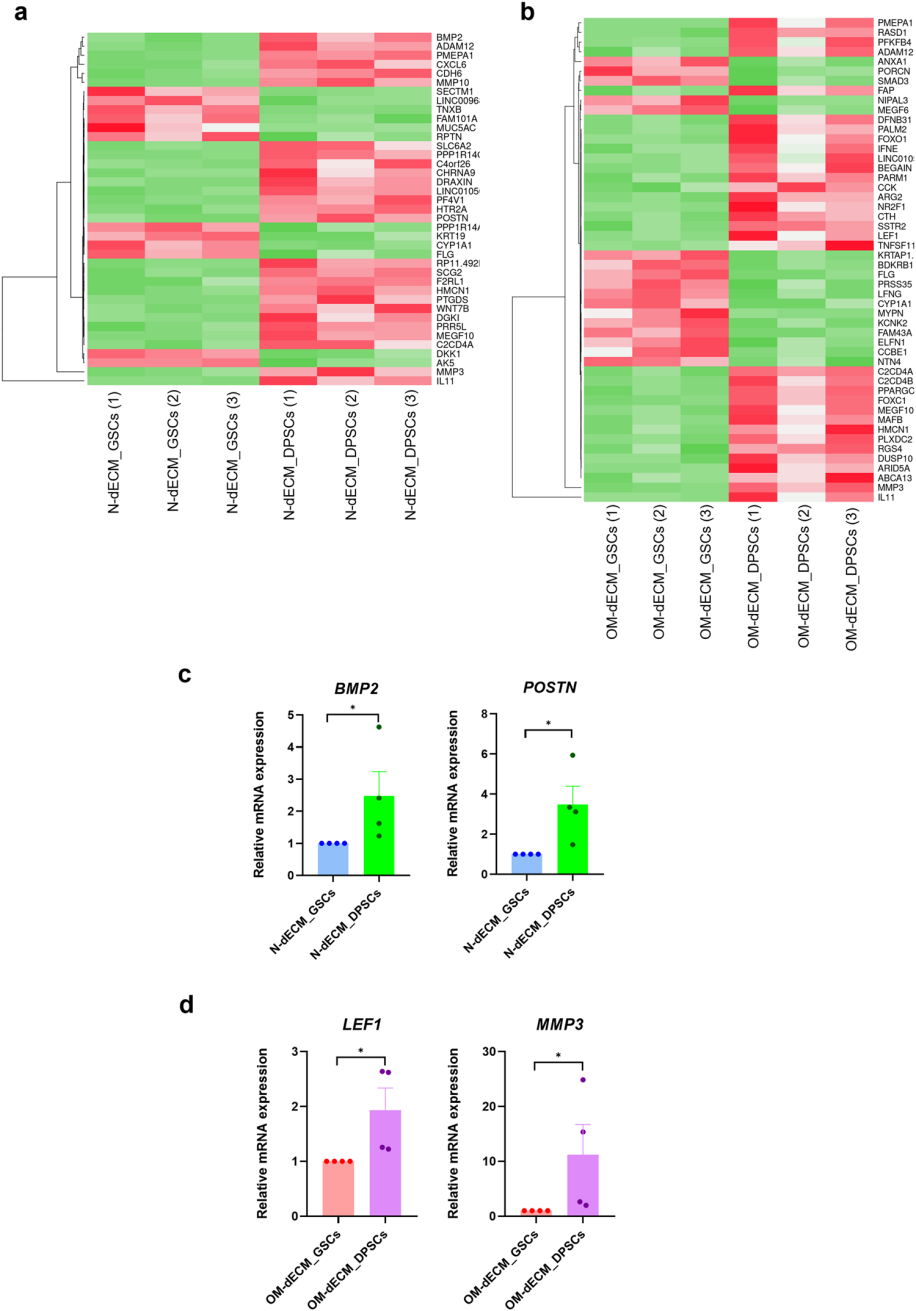
dECM_DPSCs expressed genes involved TGF-β, Hippo, and Wnt signaling pathways. The expression pattern of related genes comparing between N-dECM_DPSCs VS N-dECM_GSCs, and between OM-dECM_DPSCs VS OM-dECM_GSCs. Heatmap showed the differentially regulated genes between (**a**) N-dECM_DPSCs and N-dECM_GSCs (**b**) OM-dECM_DPSCs and OM-dECM_GSCs. Differentially expressed genes of N-dECM_DPSCs VS N-dECM_GSCs and OM-dECM_DPSCs VS OM-dECM_GSCs were validated using qPCR. The differential gene expression of (**c**) *BMP2*, *POSTN*, and (**d**) *LEF1*, *MMP3* was confirmed. Bars indicate a significant difference between groups (*p < 0.05).

GSCs were reseeded on either N-dECM from DPSCs or GSCs for 24 h, or on OM- dECM from DPSCs or GSCs for 24 h. The expression patterns comparing between N-dECM_DPSCs and N-dECM_GSCs, and between OM-dECM_DPSCs and OM-dECM_GSCs from RNAseq data were illustrated as heatmaps (Fig. 3a,b). The top 30 significantly upregulated and downregulated genes in N-dECM_DPSCs VS N-dECM_GSCs and OM-dECM_DPSCs VS OM-dECM_GSCs were listed in Table 1. Selected differentially expressed genes were validated using quantitative real-time polymerase chain reaction (qPCR), which significant upregulation of bone morphogenetic protein-2 *(BMP2)* and periostin *(POSTN)* mRNA levels was found in N-dECM_DPSCs (Fig. 3c), while OM-dECM_DSPCs significantly induced lymphoid enhancer binding factor 1 *(LEF1)* and matrix metallopeptidase 3 *(MMP3)* mRNA expression, compared with dECM_GSCs from the same condition (Fig. 3d).

**Table 1:** Top 30 differentially upregulated and downregulated genes between N-dECM_DPSCs and N-dECM_GSCs, and between OM-dECM_DPSCs and OM-dECM_GSCs.

Ensembl gene ID	Gene symbol	Gene name	LogFc	P value
Upregulated genes compared between N-dECM_DPSCs and N-dECM_GSCs				
ENSG00000211455	*STK38L*	Serine/threonine kinase 38 like	0.9864	2.55E − 18
ENSG00000138135	*CH25H*	Cholesterol 25-hydroxylase	0.9938	6.61E − 09
ENSG00000175745	*NR2F1*	Nuclear receptor subfamily 2 group F member 1	0.9974	1.59E − 12
ENSG00000144476	*ACKR3*	Atypical chemokine receptor 3	0.9985	5.30E − 09
ENSG00000149968	*MMP3*	Matrix metallopeptidase 3	1.0066	2.10E − 32
ENSG00000164251	*F2RL1*	Coagulation factor II (thrombin) receptor-like 1	1.0075	1.23E − 11
ENSG00000157680	*DGKI*	Diacylglycerol kinase iota	1.0075	4.16E − 16
ENSG00000133110	*POSTN*	Periostin osteoblast specific factor	1.0143	3.42E − 11
ENSG00000271216	*LINC01050*	Long intergenic non-protein coding RNA 1050	1.0145	7.01E − 14
ENSG00000135362	*PRR5L*	Proline rich 5 like	1.0166	1.38E − 21
ENSG00000109272	*PF4V1*	Platelet factor 4 variant 1	1.0204	8.28E − 12
ENSG00000103546	*SLC6A2*	Solute carrier family 6 (neurotransmitter transporter) member 2	1.0245	2.16E − 09
ENSG00000148848	*ADAM12*	ADAM metallopeptidase domain 12	1.0258	2.24E − 39
ENSG00000198535	*C2CD4A*	C2 calcium-dependent domain containing 4A	1.0545	4.67E − 10
ENSG00000107317	*PTGDS*	Prostaglandin D2 synthase	1.0563	8.19E − 28
ENSG00000125845	*BMP2*	Bone morphogenetic protein 2	1.1378	9.69E − 17
ENSG00000124875	*CXCL6*	Chemokine (C-X-C motif) ligand 6	1.1728	1.15E − 43
ENSG00000174792	*C4orf26*	Chromosome 4 open reading frame 26	1.1783	5.72E − 12
ENSG00000102468	*HTR2A*	5-Hydroxytryptamine (serotonin) receptor 2A G protein-coupled	1.1802	1.61E − 16
ENSG00000113361	*CDH6*	Cadherin 6 type 2 K-cadherin	1.2225	1.48E − 23
ENSG00000188064	*WNT7B*	Wingless-type MMTV integration site family member 7B	1.2393	1.20E − 13
ENSG00000198729	*PPP1R14C*	Protein phosphatase 1 regulatory (inhibitor) subunit 14C	1.275	5.23E − 14
ENSG00000124225	*PMEPA1*	Prostate transmembrane protein androgen induced 1	1.2905	5.52E − 82
ENSG00000143341	*HMCN1*	Hemicentin 1	1.3492	2.18E − 50
ENSG00000174343	*CHRNA9*	Cholinergic receptor nicotinicalpha 9	1.3689	8.15E − 19
ENSG00000095752	*IL11*	Interleukin 11	1.435	1.10E − 19
ENSG00000171951	*SCG2*	Secretogranin II	1.5984	1.30E − 53
ENSG00000166670	*MMP10*	Matrix metallopeptidase 10	1.6177	2.91E − 99
ENSG00000145794	*MEGF10*	Multiple EGF-like-domains 10	1.6768	6.98E − 45
ENSG00000162490	*DRAXIN*	Dorsal inhibitory axon guidance protein	1.7635	1.85E − 30
Downregulated genes compared between N-dECM_DPSCs and N-dECM_GSCs				
ENSG00000140465	*CYP1A1*	Cytochrome P450 family 1 subfamily A polypeptide 1	− 1.9653	8.05E − 37
ENSG00000168477	*TNXB*	Tenascin XB	− 1.3258	2.39E − 33
ENSG00000171345	*KRT19*	Keratin 19	− 1.2923	1.31E − 16
ENSG00000246430	*LINC00968*	Long intergenic non-protein coding RNA 968	− 1.1767	3.28E − 25
ENSG00000215853	*RPTN*	Repetin	− 1.1412	5.01E − 12
ENSG00000143631	*FLG*	Filaggrin	− 1.1264	3.02E − 15
ENSG00000107984	*DKK1*	Dickkopf WNT signaling pathway inhibitor 1	− 1.0694	1.01E − 13
ENSG00000141574	*SECTM1*	Secreted and transmembrane 1	− 1.0547	1.82E − 22
ENSG00000167641	*PPP1R14A*	Protein phosphatase 1 regulatory (inhibitor) subunit 14A	− 1.0504	1.65E − 13
ENSG00000154027	*AK5*	Adenylate kinase 5	− 1.0411	4.00E − 28
ENSG00000215182	*MUC5AC*	Mucin 5AC oligomeric mucus/gel-forming	− 1.0376	5.50E − 10
ENSG00000178882	*FAM101A*	Family with sequence similarity 101 member A	− 1.0179	1.38E − 09
ENSG00000082482	*KCNK2*	Potassium channel subfamily K member 2	− 0.9892	1.46E − 22
ENSG00000188581	*KRTAP1-1*	Keratin associated protein 1–1	− 0.9712	1.44E − 08
ENSG00000145681	*HAPLN1*	Hyaluronan and proteoglycan link protein 1	− 0.9422	1.15E − 15
ENSG00000107738	*C10orf54*	Chromosome 10 open reading frame 54	− 0.9407	5.27E − 15
ENSG00000166949	*SMAD3*	SMAD family member 3	− 0.9237	5.21E − 45
ENSG00000183287	*CCBE1*	Collagen and calcium binding EGF domains 1	− 0.9185	2.57E − 14
ENSG00000104725	*NEFL*	Neurofilament light polypeptide	− 0.9067	7.57E − 16
ENSG00000163661	*PTX3*	Pentraxin 3	− 0.9024	1.91E − 19
ENSG00000173641	*HSPB7*	Heat shock 27 kDa protein family member 7	− 0.8888	3.16E − 16
ENSG00000179314	*WSCD1*	WSC domain containing 1	− 0.8863	5.00E − 15
ENSG00000131737	*KRT34*	Keratin 34	− 0.8826	1.97E − 07
ENSG00000130600	*H19*	H19 imprinted maternally expressed transcript (non-protein coding)	− 0.8592	1.81E − 07
ENSG00000198910	*L1CAM*	L1 cell adhesion molecule	− 0.8575	6.50E − 11
ENSG00000123405	*NFE2*	Nuclear factor erythroid 2	− 0.8327	7.18E − 07
ENSG00000243137	*PSG4*	Pregnancy specific beta-1-glycoprotein 4	− 0.8144	5.87E − 12
ENSG00000185112	*FAM43A*	Family with sequence similarity 43 member A	− 0.8117	2.74E − 07
ENSG00000125965	*GDF5*	Growth differentiation factor 5	− 0.8107	3.47E − 07
ENSG00000127951	*FGL2*	Fibrinogen-like 2	− 0.8076	1.71E − 06
Upregulated genes compared between OM-dECM_DPSCs and OM-dECM_GSCs				
ENSG00000223414	*LINC00473*	Long intergenic non-protein coding RNA 473	1.3696	4.08E − 09
ENSG00000129422	*MTUS1*	Microtubule associated tumor suppressor 1	1.3976	3.69E − 08
ENSG00000125845	*BMP2*	Bone morphogenetic protein 2	1.4088	6.46E − 07
ENSG00000112320	*SOBP*	Sine oculis binding protein homolog	1.4169	8.41E − 08
ENSG00000169116	*PARM1*	Prostate androgen-regulated mucin-like protein 1	1.4173	5.19E − 11
ENSG00000145335	*SNCA*	Synuclein alpha (non A4 component of amyloid precursor)	1.4200	7.84E − 07
ENSG00000188064	*WNT7B*	Wingless-type MMTV integration site family member 7B	1.4207	2.35E − 06
ENSG00000124225	*PMEPA1*	Prostate transmembrane protein androgen induced 1	1.4216	2.32E − 14
ENSG00000196843	*ARID5A*	AT rich interactive domain 5A (MRF1-like)	1.4296	3.64E − 14
ENSG00000125430	*HS3ST3B1*	Heparan sulfate (glucosamine) 3-O-sulfotransferase 3B1	1.4397	1.73E − 07
ENSG00000133110	*POSTN*	Periostin osteoblast specific factor	1.4460	2.76E − 09
ENSG00000149968	*MMP3*	Matrix metallopeptidase 3	1.4676	1.10E − 22
ENSG00000271216	*LINC01050*	Long intergenic non-protein coding RNA 1050	1.4700	6.97E − 11
ENSG00000175745	*NR2F1*	Nuclear receptor subfamily 2 group F member 1	1.4759	3.72E − 12
ENSG00000138135	*CH25H*	Cholesterol 25-hydroxylase	1.5022	3.90E − 08
ENSG00000107859	*PITX3*	Paired-like homeodomain 3	1.5133	4.26E − 07
ENSG00000145423	*SFRP2*	Secreted frizzled-related protein 2	1.5327	3.04E − 07
ENSG00000140807	*NKD1*	Naked cuticle homolog 1 (Drosophila)	1.5332	1.64E − 07
ENSG00000109819	*PPARGC1A*	Peroxisome proliferator-activated receptor gamma coactivator 1 alpha	1.5474	8.42E − 22
ENSG00000180616	*SSTR2*	Somatostatin receptor 2	1.5520	8.61E − 11
ENSG00000167874	*TMEM88*	Transmembrane protein 88	1.2393	1.24E − 07
ENSG00000145794	*MEGF10*	Multiple EGF-like-domains 10	1.2750	2.58E − 15
ENSG00000184995	*IFNE*	INTERFERON epsilon	1.2905	2.42E − 11
ENSG00000054598	*FOXC1*	Forkhead box C1	1.3492	3.74E − 22
ENSG00000150907	*FOXO1*	Forkhead box O1	1.3689	1.63E − 12
ENSG00000120659	*TNFSF11*	Tumor necrosis factor (ligand) superfamily member 11	1.4350	1.72E − 10
ENSG00000138795	*LEF1*	Lymphoid enhancer-binding factor 1	1.5984	5.15E − 13
ENSG00000095752	*IL11*	Interleukin 11	1.6177	1.54E − 22
ENSG00000198535	*C2CD4A*	C2 calcium-dependent domain containing 4A	1.6768	4.91E − 30
ENSG00000205502	*C2CD4B*	C2 calcium-dependent domain containing 4B	1.7635	8.49E − 39
Downregulated genes compared between OM-dECM_DPSCs and OM-dECM_GSCs				
ENSG00000188581	*KRTAP1-1*	Keratin associated protein 1–1	− 1.880	7.37E − 11
ENSG00000140465	*CYP1A1*	Cytochrome P450 family 1 subfamily Apolypeptide 1	− 1.823	2.15E − 14
ENSG00000100739	*BDKRB1*	Bradykinin receptor B1	− 1.708	2.09E − 17
ENSG00000143631	*FLG*	Filaggrin	− 1.652	8.68E − 18
ENSG00000246430	*LINC00968*	Long intergenic non-protein coding RNA 968	− 1.605	2.82E − 08
ENSG00000205426	*KRT81*	Keratin 81	− 1.482	1.86E − 09
ENSG00000215853	*RPTN*	Repetin	− 1.458	5.05E − 09
ENSG00000185112	*FAM43A*	Family with sequence similarity 43 member A	− 1.448	5.95E − 19
ENSG00000130600	*H19*	H19 imprinted maternally expressed transcript (non-protein coding)	− 1.434	1.28E − 06
ENSG00000221852	*KRTAP1-5*	Keratin associated protein 1–5	− 1.432	1.72E − 06
ENSG00000127951	*FGL2*	Fibrinogen-like 2	− 1.418	2.16E − 06
ENSG00000173267	*SNCG*	Synuclein gamma (breast cancer-specific protein 1)	− 1.416	2.52E − 08
ENSG00000131737	*KRT34*	Keratin 34	− 1.410	2.88E − 07
ENSG00000183287	*CCBE1*	Collagen and calcium binding EGF domains 1	− 1.376	2.45E − 12
ENSG00000212724	*KRTAP2-3*	Keratin associated protein 2–3	− 1.349	3.75E − 06
ENSG00000014257	*ACPP*	Acid phosphatase prostate	− 1.349	1.56E − 07
ENSG00000115523	*GNLY*	Granulysin	− 1.342	3.14E − 07
ENSG00000146250	*PRSS35*	Protease serine 35	− 1.291	2.01E − 10
ENSG00000106003	*LFNG*	LFNG O-fucosylpeptide 3-beta-N-acetylglucosaminyltransferase	− 1.274	4.71E − 10
ENSG00000171346	*KRT15*	Keratin 15	− 1.269	1.34E − 06
ENSG00000166949	*SMAD3*	SMAD family member 3	− 1.249	6.82E − 16
ENSG00000184599	*FAM19A3*	Family with sequence similarity 19 (chemokine (C–C motif)-like) member A3	− 1.248	2.62E − 05
ENSG00000162591	*MEGF6*	Multiple EGF-like-domains 6	− 1.244	2.68E − 16
ENSG00000253230	*LINC00599*	Long intergenic non-protein coding RNA 599	− 1.233	3.85E − 05
ENSG00000225968	*ELFN1*	Extracellular leucine-rich repeat and fibronectin type III domain containing 1	− 1.224	6.20E − 12
ENSG00000074527	*NTN4*	Netrin 4	− 1.223	4.45E − 12
ENSG00000135480	*KRT7*	Keratin 7	− 1.218	2.23E − 05
ENSG00000169583	*CLIC3*	Chloride intracellular channel 3	− 1.208	3.41E − 05
ENSG00000105974	*CAV1*	Caveolin 1 caveolae protein 22 kDa	− 1.207	1.29E − 05
ENSG00000183671	*GPR1*	G protein-coupled receptor 1	− 1.203	1.97E − 06

**Figure 4:**
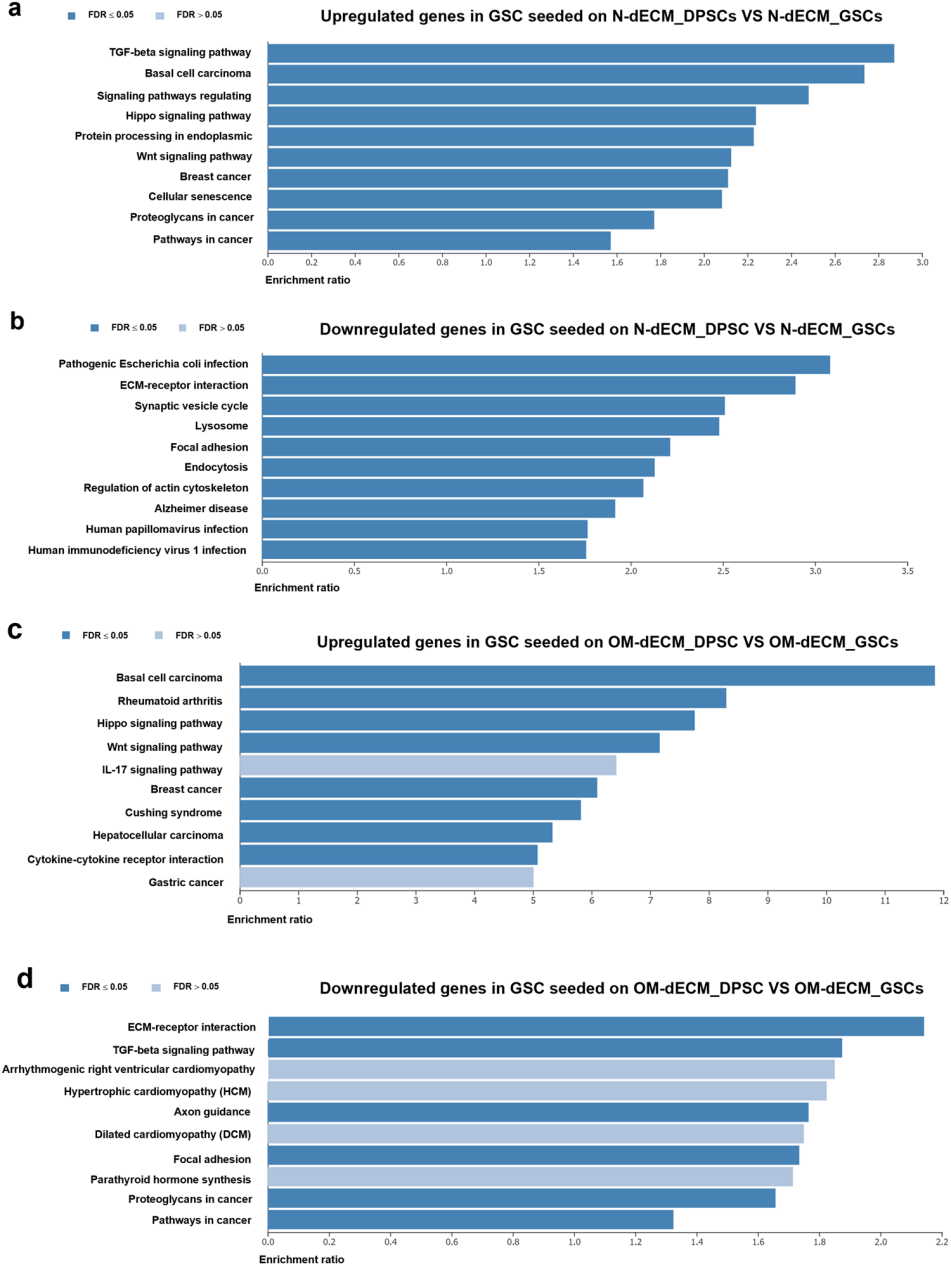
Top 10 Kyoto Encyclopedia of Genes and Genomes (KEGG) enriched pathways for (**a**) up- and (**b**) downregulated genes in N-dECM_DPSCs VS N-dECM_GSCs and (**c**) up- and (**d**) downregulated genes in OM-dECM_DPSCs VS OM-dECM_GSCs by over-representation analysis.

Bioinformatic analysis using the Kyoto Encyclopedia of Genes and Genomes (KEGG) database revealed several pathways regulated by N-dECM_DPSCs and OM-dECM_DPSCs. The upregulated genes in N-dECM_DPSCs were involved in the TGF-β, Hippo, and Wnt signaling pathways (Fig. 4a), whereas the downregulated genes were categorized in the ECM receptor interaction and regulation of actin cytoskeleton (Fig. 4b). As for OM-dECM_DPSCs, the upregulated genes were related to Hippo and Wnt signaling pathways (Fig. 4c). In contrast, the downregulated genes were found in the ECM receptor interaction and TGF-β signaling pathway (Fig. 4d).

**Figure 5:**
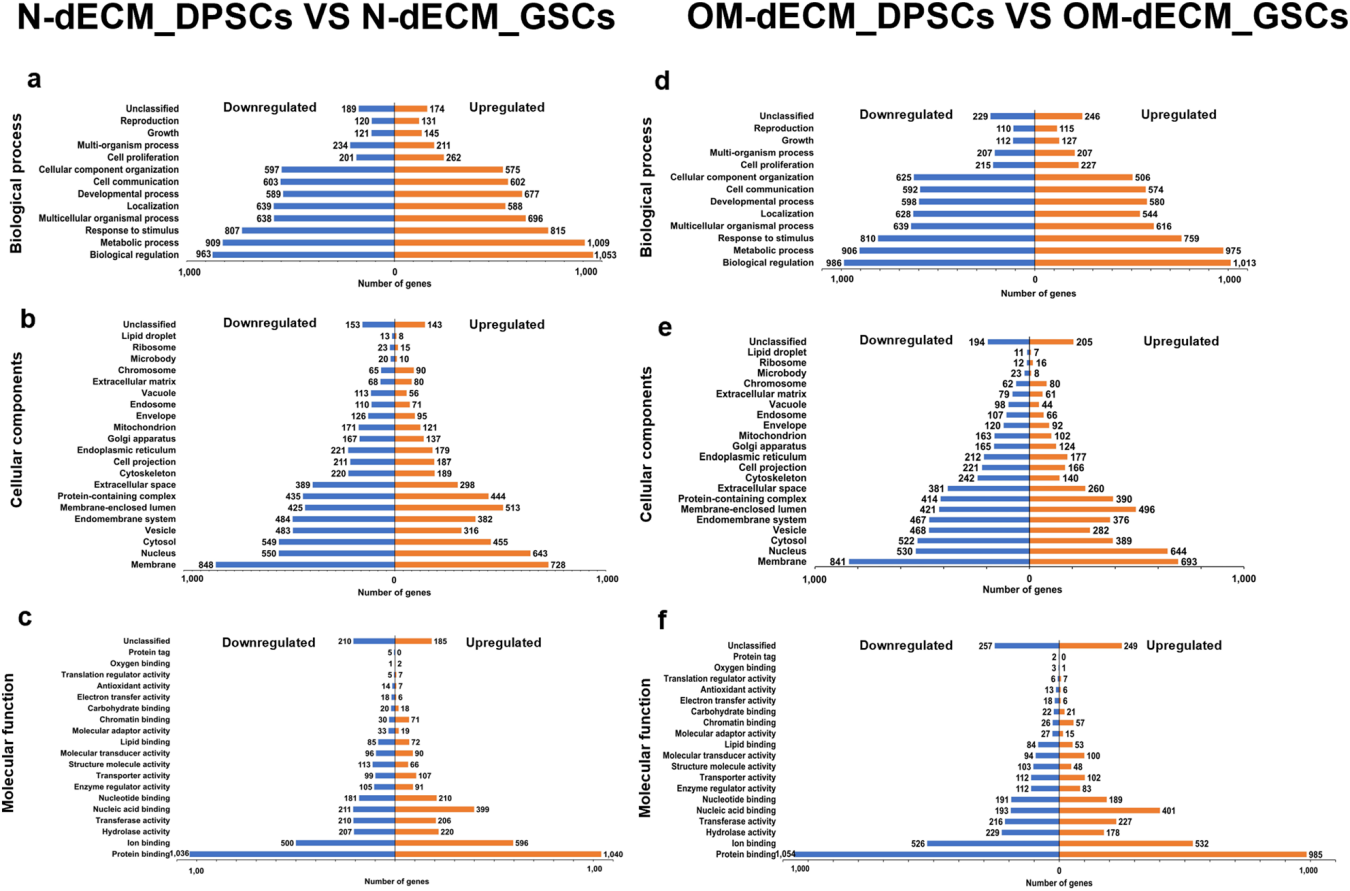
Gene ontology (GO) analyzes of the upregulated and downregulated genes comparing between (**a**–**c**) N-dECM_DPSCs and N-dECM_GSCs, and between (**d**–**f**) OM-ECM_dDPSCs and OM-dECM_GSCs. The differentially regulated genes were mainly associated with biological regulation, membrane, and protein binding in the categories of (**a**, **d**) biological process, (**b**, **e**) cellular component, (**c**, **f**) and molecular function, respectively.

Over-representation analysis was performed, and the number of genes in each gene ontology analysis for the upregulated and downregulated genes was shown in Fig. 5a–c (for N-dECM_DPSCs VS N-dECM_GSCs) and Fig. 5d–f (for OM-dECM_DPSCs VS OM-dECM_GSCs). The differentially regulated genes were mainly associated with biological regulation, membrane, and protein binding in the categories of biological process, cellular component, and molecular function, respectively.

### Characteristics and morphological appearance of ECM and dECM derived from DPSCs and GSCs

**Figure 6:**
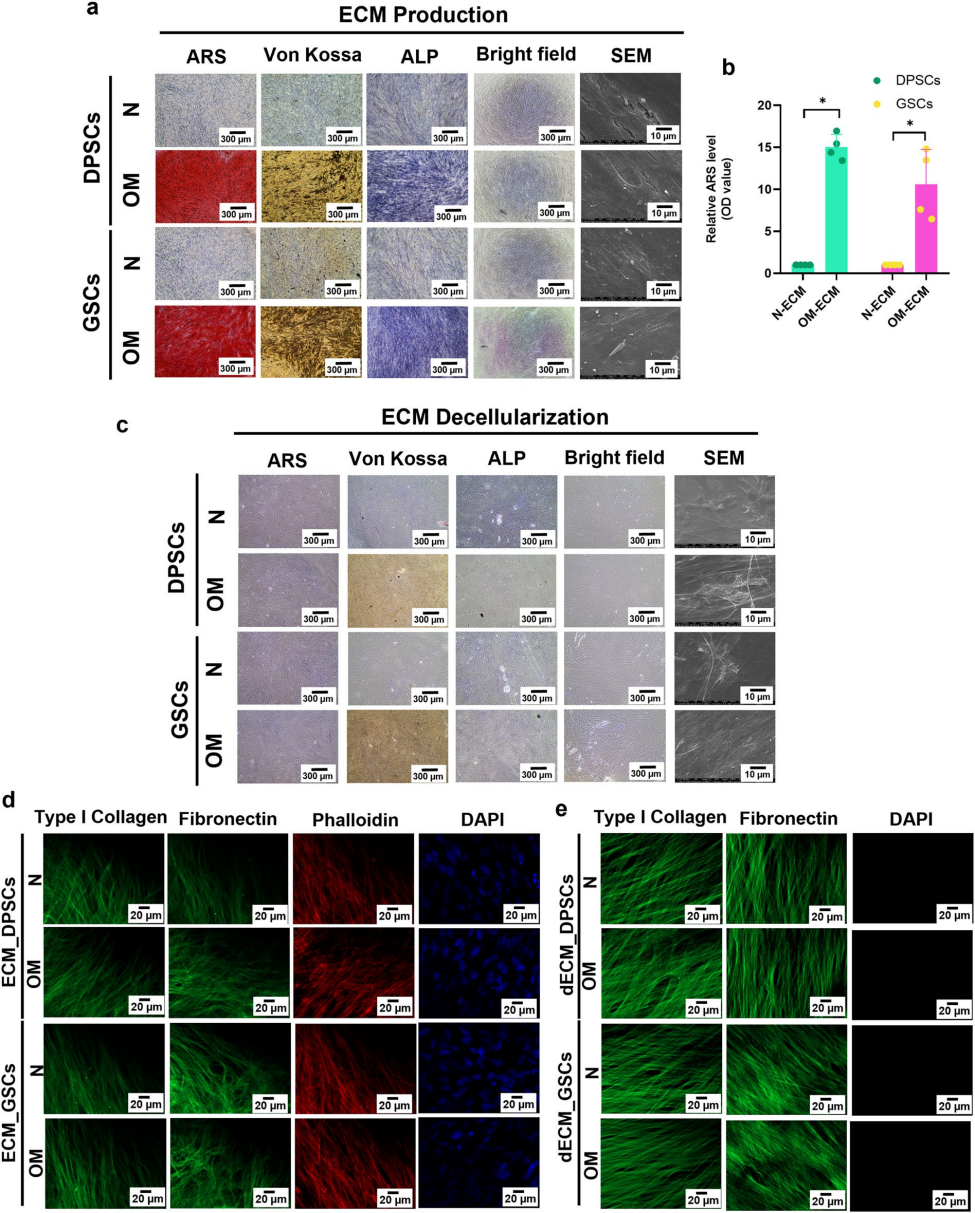
Characterization of ECM and dECM. (**a**–**c**) Morphology, mineralization, phosphate, and alkaline phosphatase (ALP) were examined. The ultrastructure of ECM and dECM was observed using scanning electron microscopic analysis (SEM). (**b**) Relative Alizarin Red S (ARS) quantification of N-, OM-ECM_DPSCs and N-, OM-ECM_GSCs. Bars indicate a significant difference between groups (*p < 0.05). (**d**) Type I collagen and fibronectin expression in ECM were determined using immunofluorescence staining. The genetic component was stained using DAPI. F-actin was visualized using phalloidin staining. **e** Type I collagen and fibronectin expression were determined in dECM. Scale bars: 10, 20, and 300 µm.

The characteristics and morphology of ECM_DPSCs, ECM_GSCs, dECM_DPSCs, and dECM_GSCs were illustrated. OM-ECM derived from both cells exhibited an increased deposit of calcium, phosphate, and alkaline phosphatase (ALP) compared with N-ECM (Fig. 6a,b). ECMs revealed fibroblast-like cells under bright-field microscope and showed similar intricate fibrillar networks observed with scanning electron microscope (SEM) (Fig. 6a). After decellularization, all dECMs were negative for ARS, Von Kossa, and ALP stainings and showed no remaining nuclei when investigated under the microscope (Fig. 6c). SEM analysis revealed dense ECM fibers after decellularization (Fig. 6c). These results confirmed that decellularization eliminated the cells and mineral deposited contents.

ECM proteins (type I collagen and fibronectin) were visualized by immunofluorescence staining. Results showed that all ECMs produced both types of ECM proteins (Fig. 6d). In addition, actin filaments and nuclei were observed (Fig. 6d). After decellularization, both ECM proteins were retained in all dECMs (Fig. 6e). Absence of actin and DAPI nuclei staining confirmed that cellular components and genetic materials were removed while ECM proteins were preserved in dECMs (Fig. 6e).

### Biological responses of GSCs on dECM_DPSCs and dECM_GSCs

**Figure 7:**
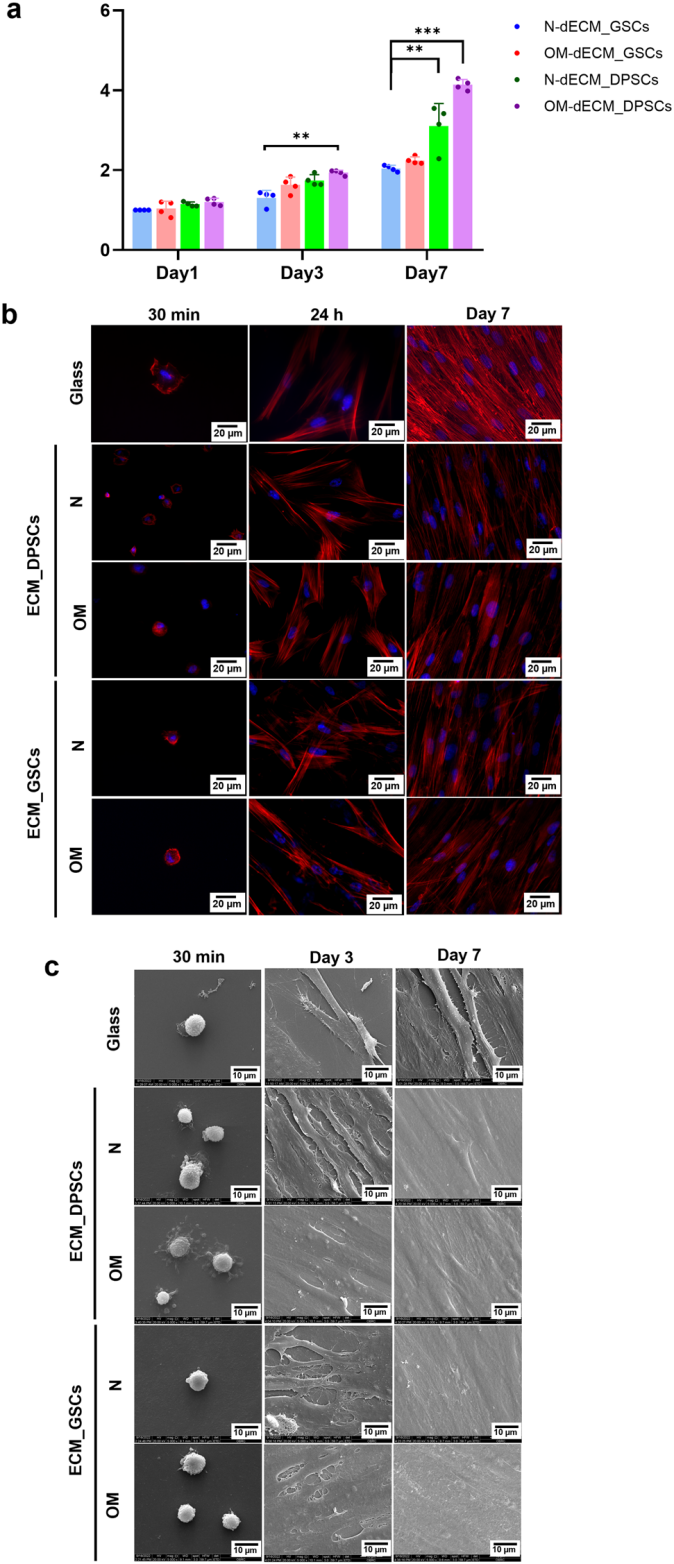
Biological response of reseeded GSCs. (**a**) Cell proliferation was determined using MTT assay. (**b**) Cellular attachment and spreading were investigated by phalloidin immunofluorescence staining. The nuclei were counterstained using DAPI. (**c**) Cell morphology was observed with SEM. Scale bars: 10 and 20 µm.

GSCs were reseeded on dECM_DPSCs and dECM_GSCs to determine the biological responses. MTT assay was employed to evaluate the GSCs cell viability on day 1, 3, and 7. GSCs that were seeded on ECM derived from GSCs and cultured in N-condition were employed as a control. GSCs were able to proliferate on all dECMs surfaces, suggesting that dECM_DPSCs and dECM_GSCs were biocompatible (Fig. 7a). Significant upregulation of GSC proliferation was found when reseeded on OM-dECM_DPSCs at day 3 and N- and OM-dECM_DPSCs at day 7 (Fig. 7a). Cell adhesion and spreading were examined after seeding GSCs on dECM_DPSCs and dECM_GSCs for 30 min, 24 h, 3 days, and 7 days. Phalloidin immunofluorescence staining was employed to visualize the organization of F-actin in the cytoskeleton. GSCs were adhered to all dECMs at 30 min after seeding (Fig. 7b). Cell spreading was initially observed at 24 h (Fig. 7b). GSCs spread extensively, and well-organized F-actin filaments were depicted on day 7 (Fig. 7b). Further, cell morphology observed with SEM and GSCs adhered to all dECMs and appeared a round shape at 30 min (Fig. 7c). However, only GSCs seeded on N- and OM-dECM_DPSCs exhibited slightly higher filopodial and lamellipodia extensions at the same time point (Fig. 7c). On day 3, GSCs were totally flattened and elongated on all dECM surfaces (Fig. 7c). GSCs were extensively spread and formed a monolayer of the cells that covered the entire surfaces on day 7 (Fig. 7c). These results suggested that both dECM_DPSCs and dECM_GSCs supported cell culture and growth in vitro, and OM-dECM_DPSCs exhibited a superior proliferative effect than those derived from GSCs.

### N-dECM_DPSCs induced mineralization via Hippo and Wnt signaling pathways, while OM-dECM_DPSCs mediated mineralization through Hippo signaling pathway

**Figure 8:**
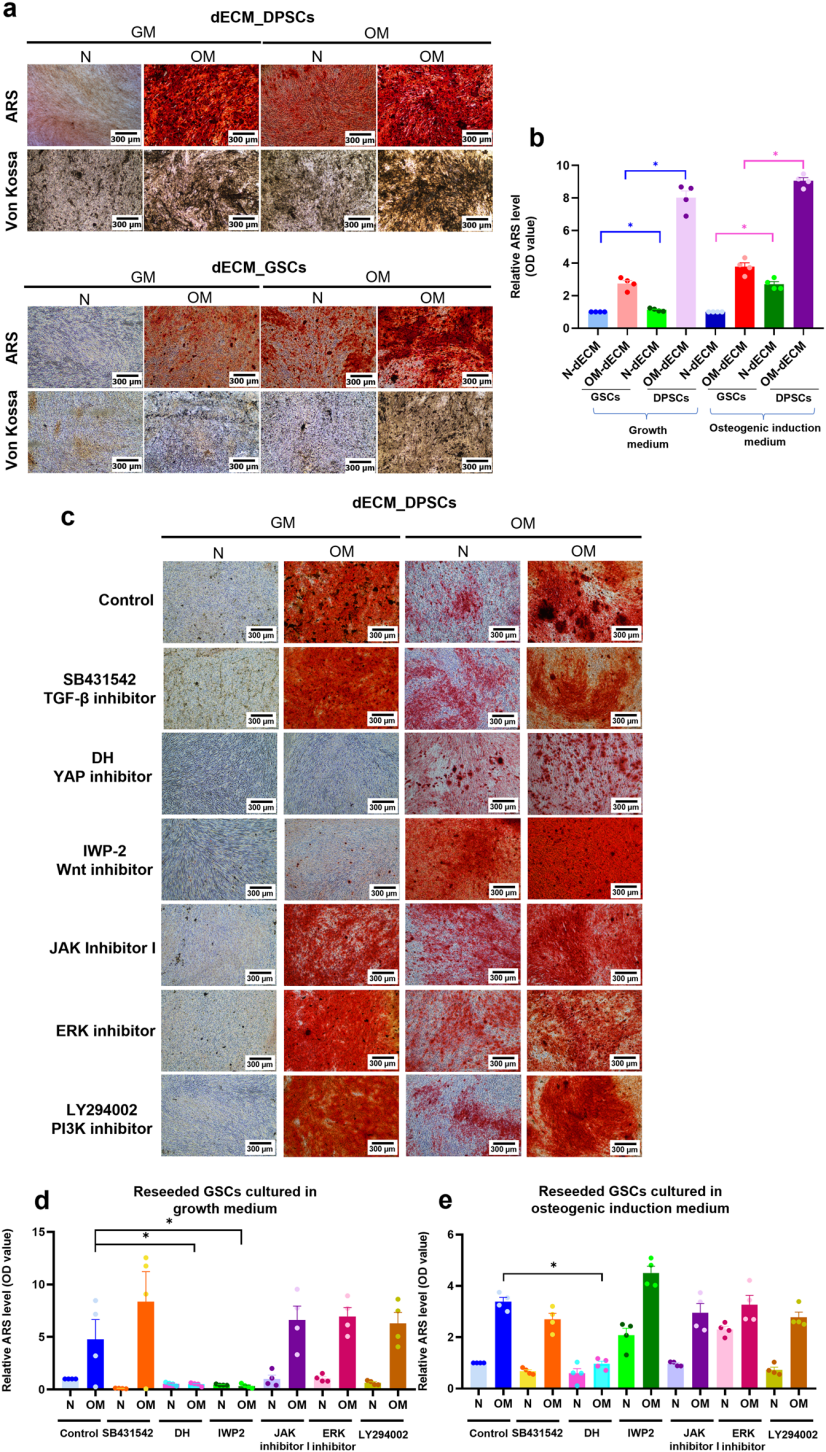
dECM_DPSCs regulated mineralization potency of GSCs via Hippo and Wnt signaling pathways. (**a**) Mineralization capacity of reseeded GSCs was determined using ARS and Von Kossa stainings. (**b**) Relative ARS quantification was demonstrated. (**c**) Effects of inhibitors of several signaling pathways were evaluated using ARS staining. Scale bars: 300 µm. (**d**) Relative ARS quantification of reseeded GSCs comparing between N- and OM-dECM_DPSCs that were cultured in growth medium. (**e**) Relative ARS quantification of reseeded GSCs comparing between N- and OM-dECM_DPSCs that were cultured in osteogenic induction medium.

To examine the osteogenic differentiation potency of GSCs on dECMs, GSCs were seeded on each type of dECMs and subsequently maintained in a growth medium or osteogenic induction medium. Mineralization was determined using ARS and Von Kossa stainings (Fig. 8a). A significant increase in GSCs mineralization capacity was observed in GSCs reseeded on N-dECM_DPSCs compared with N-dECM_GSCs and in OM-dECM_DPSCs compared with OM-dECM_GSCs (Fig. 8b, blue lines). A similar pattern was found in those cultured in an osteogenic induction medium (Fig. 8b, magenta lines). These results suggested that dECM_DPSCs enhanced the GSCs osteogenic differentiation compared with dECM_GSCs.

Based on proteomics and RNAseq results, potential regulatory pathways of dECM_DPSCs-induced osteogenic differentiation included TGF-β, Hippo, and Wnt signaling pathways. Therefore, to investigate the pathways regulated by dECM_DPSCs on reseeded GSCs, we employed inhibitors of these pathways. In addition, we targeted Janus kinase/signal transducer and activator of transcription (JAK/STAT), Extracellular signal-Regulated Kinase (ERK), and Phosphoinositide 3-kinases (PI3K) signaling in this inhibition experiment as they were downstream intracellular pathways regulated by ECM^12–14^. Therefore, we employed inhibitors of these pathways to identify the regulatory pathway of GSCs reseeded dECM_DPSCs. Results showed that YAP inhibitor (DH) and JAK inhibitor I significantly downregulated GSC mineralization on normal tissue culture surfaces (Supplementary Fig. 1a,b). Further, GSCs were pretreated with each of the cell signaling pathway inhibitors and cultured on either N- or OM-dECM_DPSCs for 14 days in a normal growth medium or osteogenic induction medium. Results showed that Wnt inhibitor (IWP-2) and DH significantly attenuated GSC mineralization after reseeding on OM-dECM_DPSCs and cultured in a growth medium (Fig. 8c,d). However, the inhibitory effect on GSC osteogenic differentiation was observed in OM-dECM_DPSCs cultured in an osteogenic induction medium after DH pretreatment (Fig. 8c,e).

**Figure 9:**
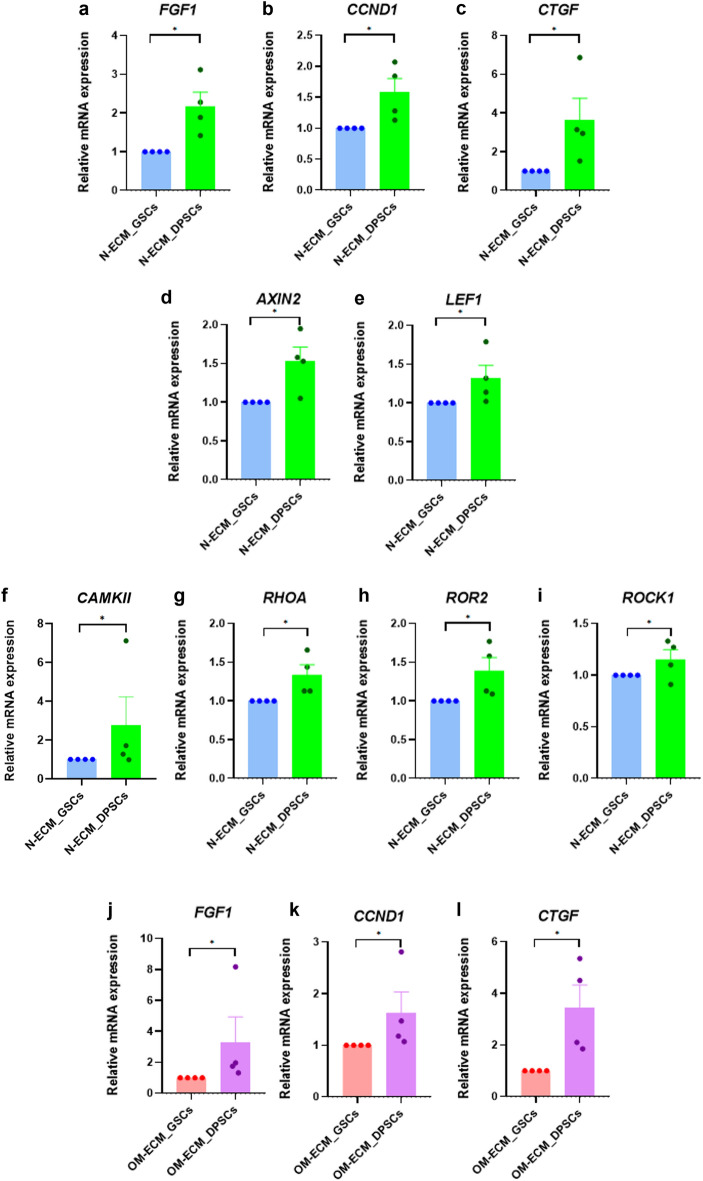
To confirm the regulatory pathways of N- and OM-dECM_DPSCs that mediated the GSC mineralization, GSCs were reseeded on N- and OM-dECM_DPSCs and N- and OM-dECM_GSCs and cultured in growth medium for 24 h. (**a**–**i**) The mRNA levels of reseeded GSCs comparing between N-dECM_GSCs and N-dECM_DPSCs. (**j**–**l**) The mRNA levels of reseeded GSCs comparing between OM-dECM_GSCs and OM-dECM_DPSCs. Bars indicate a significant difference between groups (* *p* < 0.05).

To confirm the regulatory pathways of N- and OM-dECM_DPSCs that mediated the GSC mineralization, GSCs were reseeded on N- and OM-dECM_DPSCs and N- and OM-dECM_GSCs and cultured in growth medium for 24 h. Results showed that N-dECM_DPSCs significantly upregulated genes related to Hippo signaling pathway (fibroblast growth factor 1; *FGF1,* cyclin D1; *CCND1,* and connective tissue growth factor; *CTGF*) (Fig. 9a–c), canonical Wnt pathway (axin 2; *AXIN2* and *LEF1*) (Fig. 9d,e), and non-canonical Wnt (calcium/calmodulin-dependent protein kinase II; *CAMPKII,* ras homolog family member A; *RHOA,* receptor tyrosine kinase like orphan receptor 2; *ROR2*, and rho-associated protein kinase 1; *ROCK1)* (Fig. 9f–i). In addition, OM_dECM_DPSCs significantly enhanced mRNA expression of genes related to the Hippo signaling pathway, *FGF1*, *CCND1*, and *CTGF* (Fig. 9j–l). These results confirmed that Hippo and Wnt were the major activated regulatory pathways in GSCs cultured on N-dECM_DPSCs, while OM-dECM_DPSCs regulated GSCs response via the Hippo signaling pathway.

## Discussion

The present study aims to shed light on the mineralization inductive potential of dECM_DPSCs and dECM_GSCs toward the seeded GSCs, which exhibit less capability to differentiate into osteogenic lineage^15^. Herein, we isolated ECM from different culture conditions, N and OM, to provide evidence that ECM from both osteogenic and non-osteogenic differentiation environments could enhance the osteogenic differentiation of GSCs despite the lack of osteogenic induction medium. Furthermore, we established high throughput analysis to dissect deep down into the ECM structure and functions derived from these two oral cell types.

The proteomic analysis results demonstrated that the common proteins of ECM_DPSCs and ECM_GSCs were associated with calcium ion binding. Calcium dynamics are generally mediated by the ECM components, mostly integrins and calmodulin^16^. Integrins interact with the cell cytoskeleton and send the signal to calmodulin, which acts as a calcium-binding protein, thereby initiating the intracellular calcium signaling pathways to regulate cell adhesion, proliferation, migration, and differentiation^17–19^. This finding was consistent with the results that dECM_DPSCs and dECM_GSCs supported cell proliferation and calcium deposition of reseeded GSCs. However, dECM_DPSCs induced an upregulated GSC proliferation after seeding on either N- or OM-dECM_DPSCs. In addition, OM-dECM_DPSCs significantly enhanced the mineral deposits of GSCs despite being cultured in a growth medium without mineralization chemical cues. This superior potency of dECM_DPSCs was confirmed by the signature protein analysis. DPSCs’ signature proteins related to osteogenic differentiation include TGF-β1, TGF-β2, S100A members, WNT5A, and WNT5B^20^. However, some GSCs’ signature proteins were also associated with osteogenic differentiation, for example, COL1A1, MXRA5, and TGF-β1.

The signature proteins from dECM_DPSCs and dECM_GSCs have been reported for their roles in mineralization. TGF-β1 and TGF-β2 promote early osteoblast differentiation but inhibit differentiation and mineralization in the later phases’ regeneration^21–25^. WNT5A and WNT5B, major Wnt proteins found in non-canonical Wnt signaling, have been reported for their ability to enhance osteogenic differentiation in several cells^26^. Crosstalk between Notch and Wnt signaling through WNT5A regulated the osteo/odontogenic differentiation of DPSCs^27^. WNT5B is involved in osteoblast differentiation in human BMSCs induced by phosphate^28^. Type I collagen is pivotal in supporting osteogenic differentiation. The collagen itself, or in combination with the scaffold, was promising to promote osteogenic differentiation in several cell types^29,30^. Collagen-coated plate caused spontaneous osteogenesis in amniotic membrane-derived mesenchymal stromal cells^29^. Matrix-remodeling associated 5 (MXRA5) is a member of the MXRA protein family that participates in cell adhesion and ECM remodeling^31^. The function of MXRA5 was found as TGF-β1 regulated protein and related to the chondrogenesis^32,33^. Our result showed that TGF-β superfamily found in the signature proteins of dECM derived from both cell types suggested the crucial role of TGF-β superfamily on osteogenic differentiation. However, the higher osteogenic differentiation potential of dECM_DPSCs compared with dECM_GSCs may also occur from the effect of the Wnt signaling pathway.

According to RNAseq, TGF-β signaling pathway was upregulated in N-dECM_DPSCs; however, downregulation of genes related to this pathway was found in OM-dECM_DPSCs. Despite the downregulation of genes related to TGF-β that were found in OM-dECM_DPSCs, both N- and OM-dECM_DPSCs significantly promoted calcium deposits of reseeded GSCs compared with N- and OM-dECM_GSCs in both growth medium and osteogenic medium condition, reflecting the functional property of dECM_DPSCs as the osteoinductive agent. Since TGF-β functions as both a stimulator and inhibitor of the osteogenic differentiation process, TGF-β might exert bidirectional control on osteogenic differentiation induced by dECM_DPSCs.

The RNAseq analysis emphasized the crucial role of Wnt signaling in osteogenic modulation. Based on the KEGG database, both N- and OM-dECM_DPSCs upregulated genes related to the Wnt signaling pathway. Together with proteomic data, Wnt would be the promising pathway implicated in osteogenic differentiation regulated by dECM_DPSCs. Previous studies supported that Wnt activation using small molecules in Wnt agonists enhanced odonto/osteogenic differentiation of DPSCs^34,35^. The beneficial effect of Wnt on osteogenic differentiation was also reported in dental-related stem cells^36,37^. Apart from Wnt signaling, we found that the Hippo signaling pathway was upregulated in both N- and OM-dECM_DPSCs from the RNAseq data. Previous studies highlighted the important role of the Hippo signaling pathway on osteogenesis. The transcriptional co-activators, Yes-associated protein 1 (YAP1), promoted cell proliferation and osteogenic differentiation of human periodontal ligament stem cells^38^. Hippo signaling stimulated mouse BMSC osteogenic differentiation through calcitonin gene-related peptide^39^. In addition, the upregulation of Hippo downstream effectors, including FGF1, CCND1, and CTGF, influenced osteogenic differentiation in mesenchymal stem cells^40–42^. These aforementioned studies provide evidence to support the pivotal role of Hippo signaling on osteogenic differentiation. The existence of Hippo and Wnt signaling pathways in both N- and OM-dECM_DPSCs confirmed the involvement of these pathways in osteogenic differentiation regulated by dECM_DPSCs.

To investigate the potential regulatory pathways that modulate osteogenic differentiation of dECM_DPSCs, GSCs were pretreated with several signaling inhibitors prior to reseeding on dECM and cultured in either growth or osteogenic induction medium. Results showed that IWP-2 (Wnt inhibitor) and DH (YAP inhibitor) attenuated the effect of dECM_DPSCs on GSC osteogenic differentiation under growth medium conditions. GSCs reseeded on N-dECM_DPSCs significantly upregulated genes related to Hippo and both canonical and non-canonical Wnt pathways of reseeded GSCs, emphasizing that Hippo and Wnt pathways were the major regulatory pathways mediated by N-dECM_DPSCs. However, only DH can abolish the effect of dECM_DPSC-induced osteogenic differentiation when cultured in an osteogenic induction medium. Furthermore, GSCs seeded on OM-dECM_DPSCs exhibited a significant increase of genes related to Hippo signaling, implying OM-dECM_DPSCs influenced the GSC response and osteogenic differentiation mainly via the Hippo pathway. These findings highlight the need to understand the specific signaling pathways involved in the regulation of mineralization by dECM, which may have important implications for the development of new regenerative therapies.

In summary, our study reveals that N-dECM_DPSCs promotes osteogenic differentiation via the Hippo and Wnt signaling pathways, while OM-dECM_DPSCs can mediate mineralization through the Hippo signaling pathway. We suggest that dECM_DPSCs could be developed into a promising biodegradable scaffold that provides a natural supportive structure and regenerative mineralized microenvironment, essential for tissue engineering applications. Thus, dECM represents an innovative approach toward utilizing natural and biomimetic biomaterials for tissue engineering and regenerative medicine.

## Methods

### Cell isolation and culture

**Figure 10:**
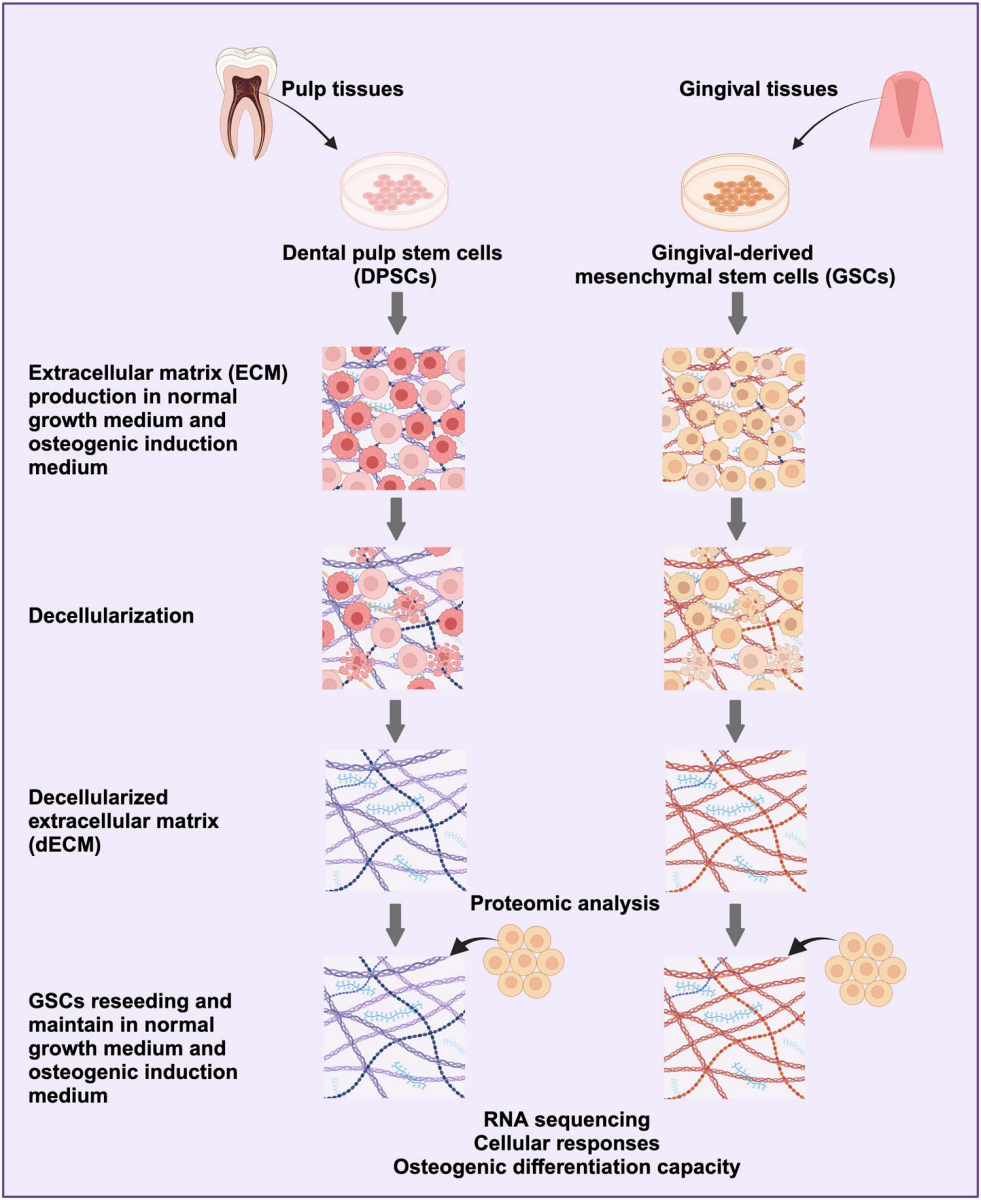
A diagrammatic representation of the experimental strategy. DPSCs and GSCs were isolated from pulp and gingival tissues collected from impacted permanent molars of healthy individuals. Cells were cultured in normal growth medium and osteogenic induction medium for 21 days to facilitate ECM production. Subsequently, decellularization was performed to obtain dECMs. A comprehensive proteomic analysis was conducted to assess the matrisome profiling of these dECMs. Subsequent experimentation involved the reseeding of GSCs onto each dECM, followed by culture in normal growth medium and osteogenic induction medium to elucidate the cellular responses and osteogenic differentiation capabilities of the reseeded cells. Furthermore, RNA sequencing techniques were employed to analyze differential gene expression, identify enriched pathways, and characterize GO terms associated with the experimental conditions. Created by biorender.com.

The study was approved by the Human Research Ethics Committee of Chulalongkorn University (approval no. 106/2022). Inform consent was obtained from study participants. Methods were carried out in accordance with the Declaration of Helsinki. Pulp and gingival tissues were collected from those tissues surgically removed according to the patient’s treatment plan. Tissues were collected from the patients who met the predefined inclusion criteria in accordance with the scientific protocol at the Department of Oral and Maxillofacial Surgery, Faculty of Dentistry, Chulalongkorn University. The inclusion criteria were as follows: healthy donors, permanent dentition, impacted molars, age range of 18–35 years, no gender specificity, and absence of tooth pathology. The explantation method was used to obtain the cells. Briefly, the collected tissues were chopped into small pieces without using enzymatic dissociation. Subsequently, the fragmented tissues were placed in 35 mm culture dishes for cells to migrate out from the tissue. The isolated cells were cultured in a growth medium composed of Dulbecco’s Modified Eagle Medium (DMEM, cat. no. 11960, Gibco, USA) containing 10% fetal bovine serum (FBS, cat. no. 10270, Gibco, USA), 2 mM l-glutamine (GlutaMAX-1, cat. no. 35050, Gibco, USA), 100 unit/ml penicillin, 100 μg/ml streptomycin, and 250 ng/ml amphotericin B (Antibiotic–Antimycotic, cat. no. 15240, Gibco, USA). The cells were incubated at 37 °C in a humidified 5% carbon dioxide atmosphere. The culture medium was changed every 48 h. The cells between passages 3 and 7 were used in the subsequent experiments. The overall experimental scheme is indicated in Fig. 10.

For inhibitory experiments, GSCs were pretreated with inhibitors for 30 min prior to reseeding on the dECM. The cell signaling inhibitors used in this study were as follows: 4 μM SB431542 (TGF-β inhibitor, cat. no. 1614, Sigma–Aldrich, USA), 20 µM dobutamine hydrochloride (DH, YAP inhibitor, cat. no. D0676, Sigma–Aldrich, USA), 25 µM IWP-2 (Wnt inhibitor, cat. No. 3533, Tocris Bioscience, USA), 3.75uM JAK inhibitor I (cat. no. 420099, Calbiochem, USA), 1.5 nM ERK inhibitor (cat. no. 328006, Calbiochem, USA), and 5 nM LY294002 (PI3K inhibitor, cat. no. A0231, Sigma–Aldrich, USA).

### Flow cytometry analysis

Surface protein expression was analyzed using flow cytometry. Single-cell suspensions were stained with fluorescence conjugated antibodies (1:50 dilution) as follows: FITC conjugated anti-human CD44 (Cat. No. 555478, BD Bioscience, USA), PE-conjugated anti-human CD105 (Cat. No. 21271054, Immuno Tools, Germany), FITC-conjugated anti-human CD90 (Cat. No. ab124527, Abcam, USA), and PerCP-conjugated anti-CD45 (Cat. No. 21810455, Abcam, USA). Mean fluorescence intensity was calculated using a FACS^Calibur^ flow cytometer (BD Bioscience, San Jose, CA, USA).

### Osteogenic differentiation

Cells (50,000 cells/well in a 24-well plate) were cultured in an osteogenic medium consisting of growth medium supplemented with 50 µg/mL ascorbic acid (cat. no. A-4034, Sigma-Aldrich, USA), 250 nM dexamethasone (cat. no. D8893, Sigma-Aldrich, USA), and 5 mM β-glycerophosphate (cat. no. G9422, Sigma-Aldrich, USA). Osteogenic differentiation potential was elucidated using ALP, ARS, and Von Kossa staining.

For ALP staining, the cells were washed with phosphate buffer saline (PBS) and fixed with 4% formaldehyde for 10 min. Then, cells were incubated with BCIP/NBT (Roche, USA) in the dark at room temperature for 30 min. The ALP-positive cells were observed using an inverted microscope (Olympus, USA).

For the ARS staining, the cells were fixed with cold methanol for 10 min and washed with deionized water. The samples were then stained with 2% ARS solution (Sigma-Aldrich Chemical) for 3 min at room temperature with gentle agitation. The mineral deposits were solubilized with 10% cetylpyridinium chloride monohydrate in 10 mM sodium phosphate. The optical density was measured at 570 nm with a microplate reader (ELx800, BIO-TEK®, United States).

For Von Kossa staining, the cells were fixed with 4% formaldehyde in PBS and further incubated with 5% silver nitrate in sterile deionized water. The samples were exposed to ultraviolet light for 5 min at room temperature. The stained cells were examined under an inverted microscope.

### Adipogenic differentiation

Cells (12,500 cells/well in a 24-well plate) were cultured in adipogenic medium comprising growth medium containing 0.1 mg/ml insulin (cat. no. 11070738 Sigma-Aldrich, USA), 1 μM dexamethasone (cat. no. D8893, Sigma-Aldrich, USA), 1 mM IBMX (cat. no. PHZ1124, Thermo Fisher Scientific, USA), and 0.2 mM indomethacin (cat. no. 53861, Sigma-Aldrich, USA) for 16 days. The intracellular lipid droplet was stained by Oil red O staining. Briefly, cells were fixed with 4% formaldehyde in PBS for 10 min, followed by incubating with 0.2% Oil Red O solution for 15 min. Lipid accumulation was examined using an inverted microscope.

### Extracellular matrix production and decellularization

The culture plate was coated with 0.2% gelatin for 2 h at 37 °C. The cells were seeded on a gelatin-coated surface and divided into two groups: N-ECM and OM-ECM. In N-ECM, cells were maintained in a growth medium for 7 days and subsequently cultured in a growth medium supplemented with 50 μg/ml l-ascorbic acid for 14 days. For OM-ECM, cells were maintained in an osteogenic medium for 21 days.

Decellularization was performed using 0.5% Triton X-100 in 20 mM ammonium hydroxide and washed with a protease inhibitor in PBS. Deoxyribonuclease A at a concentration of 0.0025% in sterile PBS was added to the samples and incubated for 30 min at room temperature for DNA removal.

### Protein extraction and digestion

Protein extraction was performed from dECM on day 21 using a Compartment Protein Extraction Kit (MERCKMillipore, USA). The protein pellets' solubilization and digestion were performed as previously described^43^. In brief, dECM pellets were solubilized in a solution containing 8 M urea, 100 mM ammonium bicarbonate, and 10 mM dithiothreitol. Cysteines were alkylated by adding iodoacetamide, and samples were deglycosylated with PNGaseF (New England BioLabs, USA, 1:100 units for 1 mg sample) and subsequently digested with trypsin/LysC (Promega, USA), at a ratio of 1:10,000 enzyme: substrate. Final digestions were done using trypsin (Worthington Biochemical Corporation, USA) at a ratio of 1:1000 (enzyme: substrate), followed by a second aliquot of trypsine/LysC (Promega, USA), at a ratio of 1:10,000 (enzyme:substrate).

### Mass spectrometry

Mass Spectrometry (LC–MS/MS) was performed as previously described^10^. In brief, chromatography was performed with an RSLCnano system (Ultimate 3000, Thermo Scientific) coupled online to a Q Exactive HF-X with a Nanospay Flex ion source (Thermo Scientific).

Peptides were trapped on a C18 column (75 μm inner diameter × 2 cm: nanoViper Acclaim PepMapTM 100, Thermo Scientific) at a flow rate of 2.5 μl/min over 4 min and subsequently separated on a 50 cm × 75 μm C18 column (nanoViper Acclaim PepMapTM RSLC, 2 μm, 100 Å, Thermo Scientific) at 50 °C at a flow rate of 300 nl/min over 211 min. MS full scans were performed in the ultra-high-field Orbitrap mass analyzer. Top 20 intense ions were further fragmented via high-energy collision dissociation activation. Ions with a charge range from 2 + to 6 + were selected for screening. Data were searched against the Homo sapiens (UP000005640) SwissProt database using Sequest HT through proteome discoverer (version 2.2). The data were subsequently processed using myProMS v3.9.3 (https://github.com/bioinfo-pf-curie/myproms) FDR calculation used Percolator. The label-free quantification was performed by peptide Extracted Ion Chromatograms (XICs) computed with MassChroQ version 2.2. To correct the XICs, median and scale normalization was applied on the total signal. For statistical analysis, a linear model was performed, and p-values were adjusted using Benjamini–Hochberg FDR procedure. Matrisome proteins database (Human Matrisome (Updated December 2022): http://matrisomeproject.mit.edu/other-resources/human-matrisome/) has been updated and used for selecting the ECM proteins out of the whole proteomics data. The mass spectrometry proteomics raw data have been deposited to the ProteomeXchange Consortium via the PRIDE (PMID: 34,723,319) partner repository with the data identifier “PXD040575” and “PXD018951” (reviewer_pxd040575@ebi.ac.uk & DYdXRjUC).

### Matrisome protein–protein interaction and enrichment pathway analysis

The significant ECM proteins detected from matrisome database were analyzed using Metascape (https://metascape.org/gp/index.html#/main/step1). PPI enrichment was determined using minimum network size = 3 and maximum network size = 500. GO enrichment analysis, categorized as a biological process, cellular component, and molecular function, was performed. The PPI figures were created by using Cytoscape version 3.9.1.

### ECM seeding experiment

GSCs were seeded at a density of 25,000 cells on dECM_DPSCs or dECM_GSCs and cultured in a growth medium for 30 min, 24 h, or 7 days. Evaluation of cell morphology, attachment, and spreading was done using SEM. Cell viability was assessed using MTT assay. For mineralization assay, GSCs were reseeded and maintained in osteogenic induction medium for 14 days.

### Immunofluorescence staining

Samples were fixed with 4% formaldehyde in PBS and incubated with 0.1% Triton-X100 in PBS. Non-specific binding was blocked with horse serum (2% v/v). Samples were stained with mouse monoclonal IgG anti-type I collagen (1:200 dilution, Abcam, UK) or mouse monoclonal IgG anti-fibronectin (1:500 dilution, Invitrogen, United States) at 4 °C overnight. The secondary antibody labeled with AlexaFluor 488 was added at a 1:2000 dilution for 2 h. F-actin organization was examined using AlexaFluor 594 Phalloidin (1:1000 dilution, Invitrogen, United States). DAPI (1:500 dilution, Invitrogen, United States) was used to counterstain the nuclei. Visualization of the target protein was detected using a fluorescent microscope with an ApoTome system (Carl Zeiss, Germany).

### Scanning electron microscopy

The samples were fixed with 3% glutaraldehyde in PBS for 30 min and dehydrated with a graded series of ethanol. Hexamethyldisiloxane was added for 5 min and the gold sputter-coat was performed for SEM analysis.

### Cell viability test

GSCs (12,500 cells/well) were reseeded on dECM. At day 1, 3, and 7, the cells were incubated with 0.5 mg/mL MTT solution (USB Corporation) for 30 min, allowing formazan crystal formation. The precipitated crystals were solubilized using a dimethyl sulfoxide and glycine buffer. The solution was measured absorbance at 570 nm by a microplate reader (ELx800, BIO-TEK®, United States). The percentage cell number was calculated and normalized with the control.

### Quantitative real-time polymerase chain reaction

Total cellular RNA was extracted using TRIzol reagent (RiboEx solution, cat. no. 301-001, GeneAll, South Korea). The cDNA was obtained by converting one microgram of total RNA using ImProm-II Reverse Transcription System (cat. no. A3800, Promega, USA). qPCR was performed using FastStart Essential DNA Green Master (Roche Diagnostic, Germany) in a CFX connect Real-Time PCR machine (Bio-Rad, Singapore). Product specificity was evaluated using melt curve analysis. The targeted mRNA expression levels were normalized to *GAPDH* gene. The relative expression was calculated using 2^−ΔΔCt^ method^44^. The primer oligonucleotide sequences are shown in Supplementary Table 2.

### High-throughput RNA sequencing

Total RNA was extracted using a RNeasy kit (Qiagen, USA). The RNA quality was examined using an Agilent 2100 BioAnalyzer (Agilent Technologies, USA), NanoDrop (Thermo Fisher Scientific Inc.), and 1% agarose gel. Library preparation was constructed using a NEBNext® UltraTM RNA Library Prep Kit for Illumina®. The constructed library was validated using an Agilent 2100 Bioanalyzer (Agilent Technologies, Palo Alto, CA, USA), and quantified by Qubit 2.0 Fluorometer (Invitrogen, Carlsbad, CA, USA). Sequencing was performed on the illumine HiSeq platform in a 2X150bp paired-end configuration. Base-calling is performed by Illumina RTA software. Demultiplexing is performed by Illumina bcl2fastq 2.17 software based on index information and the number of reads and quality score (Q30) were counted. Data were aligned to reference genome via software HISAT2 (v2.0.1)^45,46^. Differential expression analysis used the DESeq2 Bioconductor package. The sequencing data were submitted to the NCBI’s Gene Expression Omnibus (GSE226347).

### Statistical analysis

All experiments were repeated using cells derived from at least four different donors (*n* = 4). The statistical analysis was performed using Prism 8 (GraphPad Software, USA). For a two-group comparison, the Mann–Whitney U test was used. For three or more group comparisons, statistical differences were assessed using the Kruskal–Wallis test, followed by Dunn's test as a posthoc pairwise comparison. Statistical significance was considered at *p* < 0.05.

### Supplementary Information

Supplementary Information.

## Data Availability

Materials and correspondence requests should be addressed to Thanaphum Osathanon (thanaphum.o@chula.ac.th). Human matrisome proteins database has been updated and can be accessed via http://matrisomeproject.mit.edu/other-resources/human-matrisome/. The mass spectrometry proteomics raw data have been deposited to the ProteomeXchange Consortium via the PRIDE (PMID: 34723319) partner repository with the data identifier “PXD040575” and “PXD018951” (reviewer_pxd040575@ebi.ac.uk & DYdXRjUC). The RNAseq data generated in this study are available in the GEO database under accession code GSE226347. The remaining data are available within the Article, Supplementary Information, or Source Data file.
