# Supplementary material for: Human dental pulp stem cells derived extracellular matrix promotes mineralization via Hippo and Wnt pathways
Source: Sci Rep. 2024 Mar 21;14:6777. doi: 10.1038/s41598-024-56845-1 (PMC10957957; doi:10.1038/s41598-024-56845-1)
Supplement: Supplementary file 1 — Supplementary Information. [file 41598_2024_56845_MOESM1_ESM.pdf]

Human dental pulp stem cells derived extracellular matrix promotes mineralization via Hippo and Wnt pathways

Chatvadee Kornsuthisopon[1,#], Nunthawan Nowwarote[2,3,#], Ajjima Chansaenroj[1], Suphalak Photichailert[1], Sunisa Rochanavibhata[4], Nuttha Klincumhom[1], Stephane Petit[3], Florent Dingli[5], Damarys Loew[5], Benjamin P J Fournier[2,3,*], Thanaphum Osathanon[1,*]

[1]Dental Stem Cell Biology Research Unit and Department of Anatomy, Faculty of Dentistry, Chulalongkorn University, Bangkok 10330, Thailand

[2]Centre de Recherche des Cordeliers, Université Paris Cité, Sorbonne Université, INSERM UMR1138, Molecular Oral Pathophysiology, Paris 75006, France.

[3]Department of Oral Biology, Faculty of Dentistry, Université Paris Cité, Paris 75006, France.

[4]Department of Oral and Maxillofacial Surgery, Faculty of Dentistry, Chulalongkorn University, Bangkok 10330, Thailand

[5]Institut Curie, PSL Research University, Centre de Recherche, Laboratoire de Spectrométrie de Masse Protéomique, 26 rue d'Ulm, Paris 75248 Cedex 05, France.

# These authors contributed equally.

***Co-Corresponding author**

Benjamin P J Fournier, DDS, PhD

Centre de Recherche des Cordeliers, Université Paris Cité, Sorbonne Université, Inserm UMR1138, Molecular Oral Pathophysiology, 75006 Paris, France

Université Paris Cite, Dental Faculty, Department of Oral Biology, Paris, France

Tel: +33-6-955-02473

Email: Benjamin.fournier@u-paris.fr

Thanaphum Osathanon, DDS, PhD

Dental Stem Cell Biology Research Unit, Faculty of Dentistry,

34 Henri-Dunant Rd. Pathumwan, Bangkok, 10330 Thailand

Tel: +66-2-218-8885, Fax: +66-2-218-8870

Email: thanaphum.o@chula.ac.th

Supplementary Fig. 1

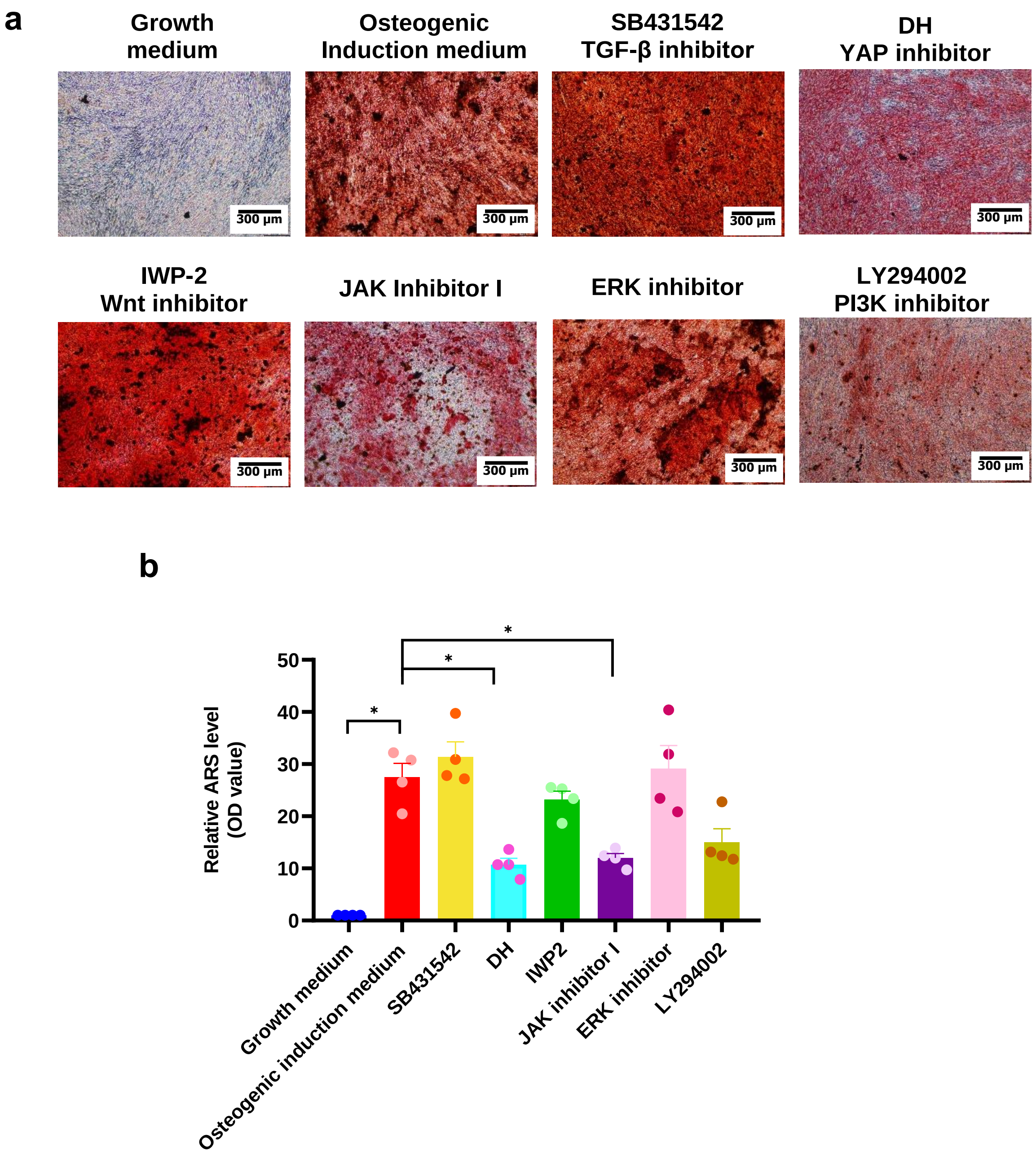

Supplementary Fig. 1. Relative ARS quantification of GSCs seeded on a tissue culture plate. GSCs were pretreated with inhibitors 30 min prior to seeding on the tissue culture surface. **a** The mineral deposition was determined using ARS staining. **b** Relative ARS quantification was illustrated. Bars indicate a significant difference between groups (* $p < 0.05$).

Supplementary Table 1. The overlapped matrisome proteins of DPSCs and GSCs

No.	Overlapped matrisome proteins of DPSCs and GSCs	Abbreviation
1	Latent-transforming growth factor beta-binding protein 2	LTBP2
2	Laminin subunit beta-2	LAMB2
3	Fibrillin-1	FBN1
4	Thrombospondin type-1 domain-containing protein 4	THSD4
5	Periostin	POSTN
6	Hemicentin-1	HMCN1
7	Nidogen-2	NID2
8	EGF-like repeat and discoidin I-like domain-containing protein 3	EDIL3
9	Fibrillin-2	FBN2
10	Tenascin-X	TNXB
11	Lactadherin	MFGE8
12	Laminin subunit beta-1	LAMB1
13	Fibulin-1	FBLN1
14	Target of Nesh-SH3	ABI3BP
15	Laminin subunit alpha-3	LAMA3
16	Adipocyte enhancer-binding protein 1	AEBP1
17	Nidogen-1	NID1
18	Laminin subunit alpha-4	LAMA4
19	Sushi, nidogen and EGF-like domain-containing protein 1	SNED1
20	Fibulin-2	FBLN2
21	Sushi repeat-containing protein SRPX	SRPX
22	EGF-containing fibulin-like extracellular matrix protein 2	EFEMP2
23	Fibulin-5	FBLN5
24	Growth arrest-specific protein 6	GAS6
25	von Willebrand factor A domain-containing protein 5A	VWA5A
26	Extracellular matrix protein 1	ECM1
27	Collagen triple helix repeat-containing protein 1	CTHRC1
28	Thrombospondin-2	THBS2
29	EGF-containing fibulin-like extracellular matrix protein 1	EFEMP1
30	Laminin subunit alpha-5	LAMA5
31	Procollagen C-endopeptidase enhancer 1	PCOLCE
32	Laminin subunit alpha-2	LAMA2
33	Microfibrillar-associated protein 5	MFAP5
34	Cysteine-rich with EGF-like domain protein 1	CRELD1
35	Slit homolog 1 protein	SLIT1
36	Protein CYR61	CYR61
37	Cysteine-rich with EGF-like domain protein 2	CRELD2
38	EMILIN-2	EMILIN2
39	Laminin subunit alpha-1	LAMA1
40	Matrix Gla protein	MGP
41	Dermatopontin	DPT
42	Tumor necrosis factor-inducible gene 6 protein	TNFAIP6

43	Latent-transforming growth factor beta-binding protein 4	LTBP4
44	Microfibrillar-associated protein 1	MFAP1
45	Fibrinogen beta chain	FGB
46	Insulin-like growth factor-binding protein 3	IGFBP3
47	Thrombospondin-4	THBS4
48	Fibroleukin	FGL2
49	Spondin-1	SPON1
50	Spondin-2	SPON2
51	Slit homolog 2 protein	SLIT2
52	Microfibril-associated glycoprotein 4	MFAP4
53	Latent-transforming growth factor beta-binding protein 2	LTBP3
54	Sushi, von Willebrand factor type A, EGF and pentraxin domain-containing pr	SVEP1
55	Connective tissue growth factor	CTGF
56	Insulin-like growth factor-binding protein 7	IGFBP7
57	Insulin-like growth factor-binding protein 5	IGFBP5
58	Vitronectin	VTN
59	Slit homolog 3 protein	SLIT3
60	Sushi repeat-containing protein SRPX2	SRPX2
61	Hemicentin-2	HMCN2
62	Cysteine-rich motor neuron 1 protein	CRIM1
63	Serpin H1	SERPINH1
64	Lysyl oxidase homolog 1	LOXL1
65	CD109 antigen	CD109
66	Inter-alpha-trypsin inhibitor heavy chain H2	ITIH2
67	Matrix metalloproteinase-14	MMP14
68	Procollagen-lysine,2-oxoglutarate 5-dioxygenase 1	PLOD1
69	Procollagen-lysine,2-oxoglutarate 5-dioxygenase 3	PLOD3
70	Cathepsin D	CTSD
71	72 kDa type IV collagenase	MMP2
72	Procollagen-lysine,2-oxoglutarate 5-dioxygenase 2	PLOD2
73	Protein-glutamine gamma-glutamyltransferase 2	TGM2
74	Prolyl 4-hydroxylase subunit alpha-2	P4HA2
75	Disintegrin and metalloproteinase domain-containing protein 17	ADAM17
76	Interstitial collagenase	MMP1
77	Lysyl oxidase homolog 2	LOXL2
78	Prolyl 4-hydroxylase subunit alpha-1	P4HA1
79	Disintegrin and metalloproteinase domain-containing protein 10	ADAM10
80	Cathepsin B	CTSB
81	ADAMTS-like protein 1	ADAMTSL1
82	Lysosomal protective protein	CTSA
83	ADAMTS-like protein 4	ADAMTSL4
84	Metalloproteinase inhibitor 3	TIMP3
85	Serine protease HTRA1	HTRA1
86	Serine protease HTRA3	HTRA3
87	Pigment epithelium-derived factor	SERPINF1

88	Glia-derived nexin	SERPINE2
89	Protein-lysine 6-oxidase	LOX
90	Disintegrin and metalloproteinase domain-containing protein 9	ADAM9
91	Cathepsin K	CTSK
92	Plasminogen activator inhibitor 1	SERPINE1
93	Cystatin-B	CSTB
94	Metalloproteinase inhibitor 1	TIMP1
95	Inter-alpha-trypsin inhibitor heavy chain H3	ITIH3
96	Alpha-2-macroglobulin	A2M
97	Cathepsin Z	CTSZ
98	Pappalysin-1	PAPPA
99	Urokinase-type plasminogen activator	PLAU
100	Serpin B8	SERPINB8
101	Serine protease HTRA1	PRSS1
102	Tissue-type plasminogen activator	PLAT
103	Lysyl oxidase homolog 4	LOXL4
104	Coagulation factor XIII A chain	F13A1
105	Cystatin-C	CST3
106	Serpin B6	SERPINB6
107	Leukocyte elastase inhibitor	SERPINB1
108	Prolyl 4-hydroxylase subunit alpha-3	P4HA3
109	Antithrombin-III	SERPINC1
110	Cathepsin L1	CTSL
111	Extracellular sulfatase Sulf-1	SULF1
112	Metalloproteinase inhibitor 2	TIMP2
113	Coagulation factor X	F10
114	Glycosaminoglycan xylosylkinase	FAM20B
115	Plasminogen activator inhibitor 2	SERPINB2
116	Bone morphogenetic protein 1	BMP1
117	Cathepsin S	CTSS
118	Inter-alpha-trypsin inhibitor heavy chain H4	ITIH4
119	Mannan-binding lectin serine protease 1	MASP1
120	A disintegrin and metalloproteinase with thrombospondin motifs 1	ADAMTS1
121	Serpin B9	SERPINB9
122	Dipeptidyl peptidase 1	CTSC
123	Lysyl oxidase homolog 3	LOXL3
124	Annexin A6	ANXA6
125	Chondroitin sulfate proteoglycan 4	CSPG4
126	Annexin A5	ANXA5
127	Galectin-3	LGALS3
128	Collectin-12	COLEC12
129	Annexin A4	ANXA4
130	Annexin A1	ANXA1
131	Plexin-B2	PLXNB2
132	Annexin A11	ANXA11

133	Galectin-1	LGALS1
134	Annexin A7	ANXA7
135	Annexin A2	ANXA2
136	Protein ERGIC-53	LMAN1
137	Glypican-1	GPC1
138	Syndecan-4	SDC4
139	Galectin-8	LGALS8
140	Annexin A3	ANXA3
141	Semaphorin-3C	SEMA3C
142	Plexin domain-containing protein 2	PLXDC2
143	Glypican-6	GPC6
144	Plexin-B1	PLXNB1
145	Complement C1q tumor necrosis factor-related protein 5	C1QTNF5
146	Plexin-A1	PLXNA1
147	C-type lectin domain family 11 member A	CLEC11A
148	Semaphorin-3A	SEMA3A
149	Syndecan-1	SDC1
150	Plexin-D1	PLXND1
151	Basement membrane-specific heparan sulfate proteoglycan core protein	HSPG2
152	Versican core protein	VCAN
153	Decorin	DCN
154	Lumican	LUM
155	Podocan	PODN
156	Fibromodulin	FMOD
157	Mimecan	OGN
158	Biglycan	BGN
159	Hyaluronan and proteoglycan link protein 1	HAPLN1
160	Protein S100-A13	S100A13
161	Host cell factor 1	HCFC1
162	Angiopoietin-related protein 2	ANGPTL2
163	Protein S100-A11	S100A11
164	Protein S100-A16	S100A16
165	Fibroblast growth factor 2	FGF2
166	Growth/differentiation factor 15	GDF15
167	Stromal cell-derived factor 1	CXCL12
168	Follistatin-related protein 1	FSTL1
169	Collagen and calcium-binding EGF domain-containing protein 1	CCBE1

Supplementary Table 2. The oligonucleotide sequences

Genes	Forward sequences	Reverse sequences	GenBank Accession Number
GAPDH	5’-TCATGGGTGTGAACCATGAGAA-3’	5’-GGCATGGACTGTGGTCATGAG-3’	NM_002046.3
AXIN2	5’-ATGATTCCATGTCCATGACG-3’	5’-CTTCACACTGCGATGCATTT-3’	NM_001363813.1
BMP2	5’-GCGTGAAAAGAGAGACTGC-3’	5’-CCATTGAAAGAGCGTCCAC-3’	NM_001200.4
CAMKII	5’-TGGGAGAAGAGGCAAAGCTA-3’	5’-AACGAAACCCTGTGGTGAAG-3’	NM_172170.5
CCND1	5’-ATCTCTGTACTTTGCTTGCT-3’	5’-AGTACATGGATATTCCCAAA-3’	NM_053056.3
CTGF	5’-TTAGCGTGCTCACTGACCTG-3’	5’-GCCACAAGCTGTCCAGTCTA-3’	NM_001901.3
FGF1	5’-GGCTTCTTCCTGCGCATCCAC-3’	5’-GGTAACGGTTAGCACACACTCCT-3’	NM_001361665.2
LEF1	5’-TCTTCCTTGGTGAACGAGTCT-3’	5’-GATGCTTTCCGTCATCGGG-3’	NM_001130714.2
MMP3	5’-ATTCCATGGAGCCAGGCTTTC-3’	5’-CATTTGGGTCAAACTCCAACTGT-3’	NM_002422.5
POSTN	5’-TGTTGCCCTGGTTATATGAG-3’	5’-ACTCGGTGCAAAGTAAGTGA-3’	NM_006475.2
RHOA	5’-TATCGAGGTGGATGGAAAGC-3’	5’-TTCTGGGGTCCACTTTTCTG-3’	NM_001313945.2
ROCK1	5’-GAAGCTCGAGAGAAGGCTGA-3’	5’-GCTCCAGTTGCAGGGTTAGA-3’	NM_005406.3
ROR2	5’-GAAGAGGACGACGACGAGGT-3’	5’-CGGGACACTGAGAGCAGAAG-3’	NM_001318204.2
